# Needs for mobile and internet-based psychological intervention in patients with self-injury and suicide-related behaviors: a qualitative systematic review

**DOI:** 10.1186/s12888-023-05477-2

**Published:** 2024-01-04

**Authors:** Meiqi Luo, Yuchuan Yue, Na Du, Yu Xiao, Chunyan Chen, Zongsu Huan

**Affiliations:** 1 College of Nursing, Chengdu University of Traditional Chinese Medicine, Chengdu, China; 2 The Fourth People’s Hospital of Chengdu, Hospital Office, Sichuan Province, Chengdu, China; 3 Psychosomatic Medical Center, The Fourth People’s Hospital of Chengdu, Chengdu, 610036 China; 4 College of Nursing, Zunyi Medical University, Zunyi, China; 5 Clinical Psychology Department, The Fourth People’s Hospital of Chengdu, Chengdu, China

**Keywords:** Self-harm, Suicide-related behaviors, Remote psychological intervention, Qualitative synthesis, Systematic review

## Abstract

**Background:**

In recent years, mobile psychological interventions have proven effective in reducing self-injury and suicide-related behaviors. Therefore, it is essential to continually enhance the user experience and address patients' needs to facilitate the development of mobile mental health interventions. Identifying patients with mobile mental health needs can be challenging for mental health professionals. To address this, we conducted a systematic review of qualitative research to synthesize the needs of patients engaged in self-injury and suicide-related behaviors for mobile and internet-based psychological interventions.

**Methods:**

This study adhered to the Preferred Reporting Items for Systematic Reviews and Meta-Analyses statement (PRISMA) and the Enhancing Transparency in Reporting the Synthesis of Qualitative Research statement (ENTREQ). We explored 11 databases and synthesized the results using thematic analysis.

**Results:**

Sixteen qualitative and mixed-method studies were included. The study found that the needs of patients with self-injury and suicide-related behaviors for mobile psychological intervention included therapy, technology, culture, privacy, communication, emotional support, personalization, and self-management. Consistent with the Technology Acceptance Model (TAM), the needs of patients with self-injury and suicide-related behaviors are influenced by the perceived ease of use and perceived usefulness of the mobile intervention. However, the findings also highlight the importance and unmet needs of peer support, communication, self-management, and empowerment in using mobile psychological interventions for patients with self-injury and suicide-related behaviors.

**Conclusions:**

Studies in this area have shown that the needs of patients with self-harm and suicide-related behaviors cover multiple stages, including basic therapeutic and technical needs and advanced emotional needs. This complexity makes it challenging to address the needs of patients engaged in self-injury and suicide-related behaviors through digital interventions. In the future, mental health professionals should be encouraged to participate in multidisciplinary collaborations to expand the use of digital interventions, enhancing remote self-management for patients and providing new strategies for the ongoing care of psychiatric patients.

We registered the review protocol on PROSPERO (CRD42022324958).

**Supplementary Information:**

The online version contains supplementary material available at 10.1186/s12888-023-05477-2.

## Background

The broad definition of self-injury encompasses both intentional self-injury and non-suicidal self-injury [1]. In this review, the term ‘suicide-related behaviors’ is used to encompass three types of the outcome of suicide: suicidal ideation, suicidal behavior, and suicidal attempts [2]. Since the purpose of this study is to explore intervention measures that prevent and address the outcome of suicide, we use the term ‘suicide-related behaviors’ instead of ‘suicide’ here. Self-injury and suicide have emerged as serious public health concerns. For example, a meta-analysis involving six countries (USA, China, Canada, South Korea, Ireland, and Australia) revealed that approximately 22% of the population had experienced suicide-related behaviors in their lifetime [3]. Another meta-analysis covering 41 countries and 597,548 participants also demonstrated that 47% of adolescents had experienced self-injury once or twice, with an overall lifetime prevalence of self-injury as high as 16.9% [4].

Psychological interventions have exhibited substantial efficacy in addressing suicide-related behaviors and self-injury [5]. There is some evidence that psychological interventions may reduce self-injury repetition and suicidal ideation. The main approaches include individual cognitive-behavioral therapy-based psychotherapy, dialectical behavior therapy, mentalization-based therapy, and group-based emotion-regulation psychotherapy [5–7]. However, traditional face-to-face psychological interventions require financial support, an adequate number of qualified therapists, and appropriate therapeutic settings [8]. Especially during the COVID-19 epidemic, due to isolation and geographical restrictions, using telephone, messages, and online interventions may be potentially better options than face-to-face psychological interventions [9]. The execution of face-to-face psychological interventions is frequently constrained by elevated costs and a fixed location, thereby fostering the advancement of psychological interventions based on mobile and internet platforms [8].

Mobile and Internet-based psychological intervention refers to the provision or support of psychological interventions through remotely connected devices, such as smartphones, wearable devices, or portable digital assistants [10]. These interventions can be considered part of the broader field of internet-based health interventions [8]. Evidence-based studies have affirmed that mobile and internet-based psychological interventions yield positive effects in reducing self-injury and suicidal ideation, along with cost-effectiveness in healthcare [8, 11, 12]. As the Internet continues to evolve, individuals involved in self-injury and suicide-related behaviors are increasingly adopting mobile and Internet-based psychological therapy [13–15].

Relevant quantitative studies have advocated for the ongoing enhancement of the availability and acceptability of mobile interventions to foster sustained patient engagement and broaden the coverage of mobile and internet-based psychological interventions [16–19]. However, we did not search for a systematic review on the need for mobile psychological interventions for patients involved in self-injury and suicide-related behaviors.

The adoption of mobile and internet-based psychological intervention has, thus far, faced challenges arising from technological difficulties, privacy concerns, and the absence of a personalized mHealth system, as indicated by pertinent qualitative research from various perspectives (including patients, families, and healthcare professionals) [17, 20–23]. Several relevant qualitative and mixed-method studies have been conducted, relying on user feedback from individuals who utilized mobile and internet-based psychological intervention products. However, the application of their findings and the methodology used are somewhat limited [24, 25]. The constraints of qualitative studies have led to insufficient interpretation and reporting of findings. This systematic qualitative review aims to enhance mental health professionals' understanding of the needs of patients engaging in self-harm and suicidal behaviors through digital interventions by providing a more comprehensive synthesis of existing research evidence.

In this study, we aim to comprehensively synthesize existing qualitative evidence to explore in depth the needs of these patients from the perspective of motivational needs, ongoing use needs, potential advanced emotional needs, etc. The research question for this qualitative synthesis was ‘What were the needs of patients engaged in self-injury and suicide-related behaviors for mobile and internet-based psychological interventions?’.

## Methods

### Protocol registration and reporting guidelines

We registered the protocol of review on PROSPERO (CRD42022324958). This study was conducted according to PRISMA and ENTREQ [26, 27].

### Data sources and search strategy

We conducted searches on 11 electronic databases using a combination of keywords and subject terms: PubMed (Medline), The Cochrane Library, Web of Science, Embase, CINAHL, ProQuest, PsycINFO, CNKI, Wan Fang, VIP, and CBM. CNKI, Wan Fang, VIP, and CBM are prominent electronic databases in China commonly utilized for retrieving Chinese literature in meta-analyses. The electronic databases were searched from their inception until April 2022, with no restriction on the year of publication.

To ensure a comprehensive search, we explored the reference lists of eligible publications to identify additional relevant publications. The keywords and subject terms employed in the search encompassed terms such as self-harm, self-injury, deliberate self-harm, non-suicidal self-injury, self-burn*, self-mutilation, self-cutting, suicide*, mobile health, text message, eHealth, mHealth, mobile intervention, mobile-based intervention, mobile apps, apps, computerized, online self-help, online intervention, online therapy, web therapy, web intervention, and Information Communication Technology. The complete search strategy is available in Additional file 1.

### Eligibility criteria

The inclusion criteria were as follows: (a) population: patients at risk of or with a history of self-injury or suicide-related behaviors, including those with a documented history and diagnosis of suicide-related behaviors or self-injury; (b) phenomena of interest: the needs of patients engaging in self-injury and suicide-related behaviors concerning mobile and internet-based psychological interventions; (c) mobile and internet-based psychological interventions: these encompass psychological interventions delivered through mobile applications and web-based tools; (d) data: ​qualitative interview texts available for extraction; (e) study design: qualitative studies and mixed-method studies; (f) languages: written in English and Chinese.

The exclusion criteria are as follows: (a) population: patients whose symptoms are not primarily characterized by self-injury or suicide-related behaviors; (b) study design: quantitative research; (c) mobile and internet-based psychological interventions: ​interventions conducted solely through phone calls; interventions where the primary aim is not to address self-injury or suicide-related behaviors; (d) data: descriptive texts lacking the patient's own experience, or mixed studies where the patient's description content cannot be extracted separately; (e) other: inability to extract qualitative data from the original text, studies reported solely as abstracts, or studies for which we cannot obtain full-text copies; repeatedly published studies.

### Screening process for data

All potential articles underwent review by four reviewers trained in evidence-based research, following a two-stage selection process based on inclusion criteria [28]. Initially, two reviewers conducted literature screening, focusing on titles and abstracts to eliminate duplicates using the literature management software, EndNote, and exclude references clearly irrelevant to the topic. In cases where the conclusions of the two reviewers were inconsistent or both indicated difficulty in judging alignment with inclusion and exclusion criteria based on abstracts, such references were deemed uncertain. Any uncertain references and those with abstracts or titles meeting the inclusion criteria proceeded to the full-text review. In the second stage, another two reviewers independently read the remaining works. Any questions or concerns about the articles were forwarded to a third reviewer for clarification. Moreover, any identified incorrect or missing information was communicated to the authors. Articles lacking a timely response (within two weeks) were considered for deletion due to insufficient information [29, 30]. The author's response, if provided, would serve as a supplement to the content of the reference. Ultimately, all articles were assessed based on uniform inclusion and exclusion criteria to ensure consistency.

### Data extraction

Before formally extracting the data, we conducted a pre-extraction of data from 1–2 articles using NVivo software. This software allows for data extraction from articles and multi-person collaboration to ensure that each researcher is familiar with the data extraction process. Two researchers separately retrieved each portion using the NVivo software, and all extracted data were then validated by a joint discussion among the study members.

Two distinct sets of data, one containing participant quotations and the other providing a summary of the findings, were extracted from the included literature. The researcher distilled data from the articles to ensure that the review findings were based solely on the authentic experiences of the participants [30]. Finally, our research team collaborated to assign a level of credibility to the extracted results [31]. We categorized credibility into three classes: unequivocal, credible, and unsupported. Unequivocal results (U) were those supported by participant quotations. Credible findings (C) were substantiated by illustrations that featured researchers’ summaries based on quotes rather than direct participant quotes. Unsupported findings were those not supported by the data [32]. No unsupported findings were identified in this study. The complete data findings and credibility ratings are available in Additional file 2.

### Quality assessment

Using the Critical Appraisal Skills Program (CASP), we assessed the methodological quality of the qualitative articles included in the review [33]. CASP contains ten questions, which need to choose *yes*, *no*, or *can’t tell*. First, two reviewers independently extracted the raw data and the evaluation through NVivo, a collaboration software that extracts information from the original text and annotates it. The extracted data included study purpose, value, recruitment methods, inclusion criteria, study design, data analysis, conclusions, etc. Next, two researchers independently coded and annotated the articles about ten evaluation questions in NVivo and chose *yes*, *no*, or *can’t tell* for every question. Before formally extracting the data, we conducted pre-experiments on data extraction for 1–2 articles to ensure that each researcher could proficiently work with the software and extract data according to their ideas. If there were disagreement, the study members would have discussed it. The CASP is part of the assessment of the credibility of the results of each review and therefore we did not use the criticality assessment as a basis for exclusion.

### Data analysis

We utilized thematic analysis for meta-synthesis [34]. The thematic analysis comprised three stages, each independently completed by two researchers. Final decisions were reached through group discussion. The first stage was the free encoding of the already extracted structural data from the first step of the quality assessment in NVivo, line by line. During the encoding process, we would check the meaning and data of contexts. The second stage reorganized the codes into relevant categories, each has been analyzed for adequacy. The third stage involves examining each category and comparing it to others while specifically looking for similarities and differences. Similar categories are merged into higher-level themes, moving beyond the results of the original research into a higher level of elaboration of the phenomenon. We used the Confidence in the Evidence from Reviews of Qualitative research (GRADE-CERQual) approach to evaluate the degree of confidence in the findings of systematic reviews of qualitative research (or qualitative evidence synthesis). Qualitative evidence synthesis confidence is an assessment of the degree to which a review finding is an accurate representation of the phenomenon. Therefore, it is different from the quality assessment conducted in the initial literature screening, and the review findings rated as low confidence means it is possible that the review findings reasonably reflect the phenomenon of concern [35]. For this purpose, we did not remove the presentation of low-quality evidence. The GRADE-CERQual included four components for assessing how much confidence to place in findings from qualitative evidence syntheses: methodological limitations, coherence, adequacy of data, and relevance [35].

## Results

### Search results

**Fig. 1:**
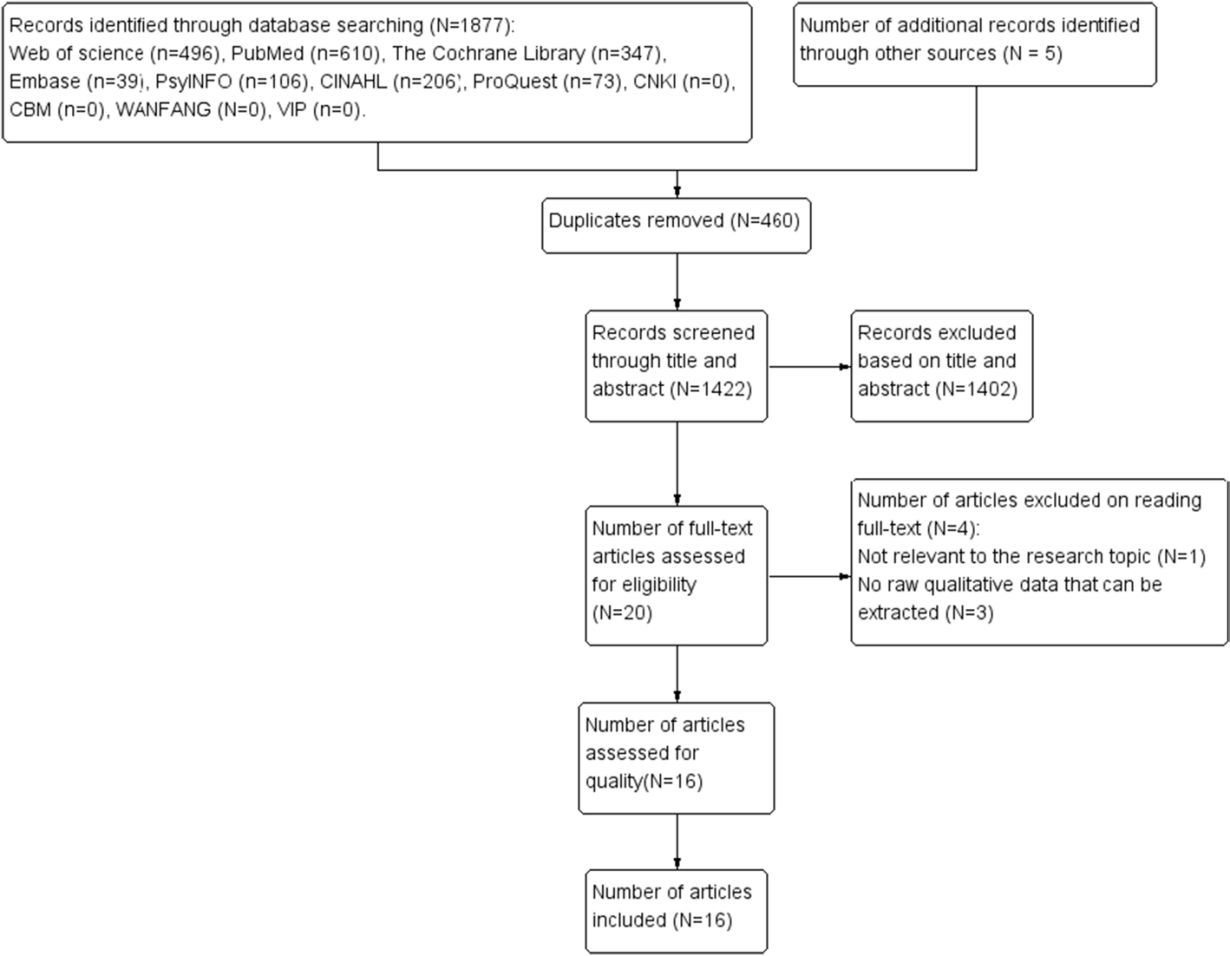
Flow diagram for study selection: PRISMA

This systematic evaluation found 1877 articles in the initial search, and the researcher's manual searching included five relevant studies. After reading the abstracts and titles, we removed 460 duplicates and 1402 studies that did not match the inclusion criteria. We reviewed all 20 of the remaining articles in their entirety and eliminated four of them: one paper with irrelevant research topics and three with unextracted primary qualitative data. Eventually, we included 16 articles. Figure 1 illustrates the flow diagram for study selection using PRISMA [36].

### Study characteristics

The sixteen articles' study characteristics were shown in Table 1. There were twelve qualitative studies and four mixed-method studies. Eleven studies used semi-structured interviews, three used open-ended statements, and two were conducted using focus groups. Fourteen articles used thematic analysis to refine the research, one used grounded theory, and one used inductive qualitative content analysis in the included literature. The mixed-method studies involved 488 participants, and the qualitative study involved 169 participants (we only extracted data from participants, excluding data from family members, medical staff, and service providers). Among the included studies, five studies focused on adolescents, one study focused on college students, and two studies focused on veterans. The age range of the participants was from 12 to 65 years. Seven studies mainly used smartphone apps for online interventions, three attempted to combine online interventions with offline professional help, and six used development web pages or online tools to deliver interventions. Seven regions were covered: Australia, the USA, the UK, Austria, Canada, Sweden, and the Netherlands.

**Table 1:** Study characteristics for final inclusion in the articles

**Author /Date/Country**	**Sample recruitment method**	**Sample (number/gender/age)**	**Paradigm / Methodology**	**Type of mobile and internet-based psychological intervention**	**Data collection/Analysis methods**	**Topic / Aim / Research questions**	**Key findings**	**Limitation**
Anja Cuš ˇ 2021 Austria [20]	A purposive sampling approach at a Department of Child and Adolescent Psychiatry	*N* = 15/Female adolescents/Aged 12 to 18 years	Qualitative interview study	Smartphone apps	The semi-structured interviews/A reflexive thematic analysis approach by combining both con- structivist and experiential approaches	1) Explored the needs of young people in relation to their everyday experiences of managing NSSI and their preferences for smartphone interventions. 2) Young people's perceptions of how technology itself can improve or hinder participation in these interventions	Mental health condition, person and technology	The study findings would be strengthened by inclusion of male and/or gender diverse participants
Craig Mackie 2017 Canada [37]	Emergency department clinical staff at The Ottawa Hospital were asked to refer adult men to the research team who met the eligibility criteria	*N* = 6 men/Aged 19 to 41 years	Qualitative study	Smartphone app combined with face-to-face therapy	Semis-structured qualitative interviews/A thematic, grounded theory approach	To develop a treatment manual and to describe experience of receiving and delivering a novel blended therapy combining a customized smartphone application	Trust and connection	1) Small sample size. 2) The study use of a beta version of the app so bugs in the program have more prominence than would be expected in a final version
Olivia Simonsson 2021 Sweden [21]	Patients who had participated in the quantitative part of the pilot trial of online emotion regulation individual therapy for adolescents (ERITA) in Stockholm, Sweden	*N* = 9/6 females and 3 adolescents as nonbinary/Aged 14–17 years old	Qualitative study	Online emotion regulation individual therapy: a mobile app and online course	Semi-structured interviews/Thematic analysis	This study aims to explore the experiences of a novel online treatment for adolescents with NSSI and their caregivers	1) Theme support can come in different shapes. 2) Self-responsibility can be empowering as well as distressing. 3) Acquiring new skills and treatment effect	1) Sampling from a single pilot study limits the transferability of the results. 2) Some families were interviewed months after ending the treatment possibly introducing recall bias
Ozlem Eylem 2021 Netherlands [17]	Patients recruited from the general population using social media and newspaper advertisements	*N* = 12/9 women and 3 men/Aged 23 to 56 years	Mixed -methods study	Online self-help intervention and online guidance from the coaches	Telephone interviews which have an open question and a topic guide/Thematic analysis	The objectives of this study are twofold: 1) To investigate the feasibility of the adapted online intervention among Turkish migrants in the UK and in the Netherlands; 2) To investigate the likely effects of the culturally adapted online intervention in reducing suicide-related behaviors when compared with the treatment as usual	1) Theme 1: Therapeutic change Therapeutic alliance. 2) Theme 2: The gap between ‘reading it’ and ‘doing it’ in real life Feeling connect. 3) Theme 3: Recommendations for improvement More diversity	1) Lack of anonymity during the recruitment process. 2) Small sample size might be another important barrier
J. Kasckow 2014 United States [38]	Recruitment took place at the VA Pittsburgh Healthcare System Psychosis Service	*N* = 10 veterans/Not mentioned/mean of age 57.40 years	Qualitative study	Telehealth dialogues	Open-ended statements/Thematic analysis	1) To test an improved tele-health system for monitoring suicidal patients with schizophrenia. 2) To obtain patients' sense of experience and opinions	1) Certain topics elicited strong emotional responses. 2) There were concerns with confidentiality. 3) Some content was too vague. 4) There were problems with vocabulary and wording	Not mentioned
McManama O’Brien 2019 United States [39]	Patients be recruited from the inpatient psychiatric unit of an urban general hospital in the northeast United States	*N* = 8 Adolescents/Not mentioned/Aged 13 to 18 years	Qualitative interview study	Mobile health (mHealth) tools	Semi-structured interviews/Thematic analysis	The purpose of this study was to gather feedback to improve a brief alcohol intervention provided to suicidal adolescents during psychiatric hospitalization, and to develop a mHealth tool to extend care after discharge	Feedback on mHealth booster of in-person component, modalities, content and functionality, frequency and general thoughts	Limitations on sample size and representation
Tobias Schiffler 2022 Austria [40]	Using convenience sampling, patients from a transitional psychiatric ward at the hospital of Hietzing in Vienna, Austria	*N* = 13/12 women and 1 man/ Aged 18 to 23 years	Qualitative study	A mobile dialectical behavior therapy app	Individual semi-structured interviews/Thematic qualitative text analysis	The aim of the present study was to investigate whether using a mobile app with DBT content for 30 days influenced the perception of suicide-related behaviors and NSSI in TAY with BPD and how such an app could benefit this targets group	1) Experiences with DBT skills 2) Phenomenon of self-injury 3) Feelings connected with self-injury 4) Dealing with disorder-specific symptoms 5) Prevention of self-injury 6) Attitude toward skills apps	1) The small amount of time spent using the app had a substantial impact upon the data collected. 2) There was an imbalanced distribution of gender. 3) Limited time resources available
J. Kasckow 2015 United States [41]	Patients recruited from the VA Pittsburgh Healthcare System	*N* = 9 veterans/Not mentioned/Mean of age 57.0 years	Qualitative study	Telehealth dialogues	Open-ended statements/Thematic analysis	The purpose of this presentation is to describe the process we undertook. We also compared responses in these participants with a history of major depression to those given by a group of consumers with schizophrenia	(1) Certain topics elicited too strong of an emotional response; (2) There were concerns with confidentiality of responses; (3) There were problems with vagueness of questions	Not mentioned
Bethany Cliffe 2022 United Kingdom [24]	Participants were students from one UK University. Adverts around campus and on social media provide links to an online information sheet and form to register interest	*N* = 25 university Students/20 female, 4 male and 1 nonbinary/Aged 18 to 31 years	Qualitative study	Smartphone app	Semi-structured interviews/An inductive qualitative content analysis	This study sought to assess the acceptability of a smartphone app called Blue Ice for university students who self-injury	1) The content of Blue Ice. 2) The use of Blue Ice with university students. 3) The function of Blue Ice. 4) Comparison with other support. 5) The implementation and uptake of Blue Ice	Because COVID-19 lockdown, participants were unable to use the app which may have limited their understanding of it and influenced their feedback. This study may also be limited by a lack of diversity
Natasha Josifovski 2022 Australian [42]	Patients come from Australian emergency department in The Royal Prince Alfred Hospital, Sydney, and Toowoomba Hospital, Queensland	*N* = 6/Not mentioned/Aged 16–65	Mixed- methods study	A text message and online brief contact intervention	Semi-structured interviews/Thematic analysis	This study investigated the feasibility and acceptability of the RAFT brief contact intervention	1) Text messages served as a reminder for the participant to focus on their mental health and included useful tips to improve their mental state. 2) The text messages were often a comfort in knowing that someone was there and that someone cares	This was a pilot study with a small, almost all female sample and no control group, with difficulties identifying eligible participants in the clinical setting
Candice Biernesser 2021 United States [23]	Adolescent come from an intensive outpatient program at an academic medical center in Pennsylvania	*N* = 15/7 female, 5 male, 2 of whom reported other gender identities and 1 not described/Aged 13 to 17 years	Qualitative study	A tool about automated monitoring of digital media use	Focus groups with adolescent/thematic analysis approach	This study examines the current context of digital media monitoring for adolescents engaged in suicide-related behaviors seeking clinical care to inform the need for automated monitoring and the factors that influence the acceptance of automated monitoring of adolescents engaged in suicide-related behaviors’ DMU within clinical care	1) There should be a balance between the need for protection and free expression and privacy. 2) Perceptions on automated digital media monitoring: protection from harm; automated risk detection; Involvement of clinicians; Loss of digital privacy; Potential for false labeling; Tendency to alter behavior; Communication with parents about risk	First, the use of a convenience sample, the small sample size, and the exploratory nature of this study limit our ability to generalize our results to a larger population. 2) There was a lag in data collection
E. Baileyy 2021 Australia [22]	Participants were current clients of a youth mental health clinic in Melbourne, Australia	*N* = 15/9 female, 5 male, and 1 as transgender/Aged 17 to years	Qualitative study	A closed website incorporating 3 key components: therapeutic content delivered via comics, peer-to-peer social networking, and moderation by peers and clinicians	Semi-structured interviews/Inductive thematic analysis and consensual qualitative research method	The aim of this study is to report qualitative data collected from study participants regarding their experience of the web-based social network and the consequent safety features	1) A safe and supportive environment. 2) The importance of mutual experiences. 3) Difficulty engaging and connecting. 4) The pros and cons of banning discussions about suicide-related behaviors	1) Small sample size. 2) Qualitative methods bring a degree of researcher subjectivity. 3) They did not analyze relationships between the themes. 4) Participants were not asked to review the transcripts or the study findings
Rebecca Grist 2018 United Kingdom [43]	Patients are recruited by all clinical teams at Oxford Health NHS Trust	*N* = 33/ Participants were predominantly girls/ Aged 12 to 17 years	Qualitative study	A mobile phone app	Semi-structured interviews/Thematic analysis	The aim of this study was to explore the acceptability, use, and safety of, a mobile phone app for young people who self-injury and who are attending child and adolescent mental health services	1) Appraisal of the app. 2) Usability of the app. 3) Safety. 4) Benefits of the app. 5) Agency and control. 6) The app less helpful	1) The sample is mainly young people who are actively participated and may be more likely to present a positive attitude. 2) Missing explanation for interrupted visits to personnel. 3) Some of the questions in the post use interview maybe subject to recall bias. 4) The study design may be possible synergic effects
Ana Radovic 2021 United States [44]	Participants were recruited through primary care clinics and flyers posted in clinical settings at Pennsylvania	*N* = 11 adolescents/5 female and 6 male/ Aged 15 to 17 years	Qualitative study	A decision support tool: screening wizard	Interviews and focus groups/Codebooks were inductively developed based on the content and Completed coding was used to produce thematic analyses	This study aims to describe multi-stakeholder perspectives of adolescents, parents, and providers to understand the potential barriers to the implementation of a technology-based decision support tool	Theme 1: Adolescents believe that depression screening should occur in pediatric primary care; Theme 2: There is concern that accurate self-disclosure does not always occur during depression screening; Theme 3: SW is viewed as a tool that could facilitate depression screening and that might encourage more honesty in screening responses. Theme 4: Adolescents do not want SW to replace mental health discussions with providers. Theme 5: Providers want to maintain autonomy in treatment decisions	1) Limitation is that the study did not collect information about gender or sexual minority status. 2) Small sample size and diversity
Joseph Tighe 2020 Australia [25]	Participants were purposefully selected due to their close proximity to the research team in Broome, Western Australia	*N* = 13/10 identified as female and 3male/Aged 19 to 29 years	Mixed methods study	A suicide prevention app	Semi-structured interviews/Thematic analysis	This paper explores the pilot use and acceptability data for the iBobbly suicide prevention app	1) Acceptability — being private and acceptable 2) Cultural appropriateness and future help-seeking 3) Helping with feelings and creating distractions	With a small sample and usage data included from just the intervention group, the analyses lacked power and precision
Mareka Frost 2016 Australia [45]	Participants were recruited to complete an Internet survey via a variety of online and offline sources in Australia	*N* = 457/Not mentioned/Mean age of 18 years	Mixed methods study	Provide online help for information	Open question/Thematic analysis and an inductive approach	To investigate the perspectives of young people who self-injure regarding online services, with the aim of informing online service delivery	1) Information 2) Guidance 3) Reduced isolation 4) Online culture 5) Facilitation of help-seeking 6) Access 7) Privacy	Recruiting was not randomized which may have led to a sample that is not representative. There was a high percentage of female participants

### Methodological quality and dependability of studies

The content of the CASP is shown in Table 2.

**Table 2:** Quality assessment

Serial number	Title	Q1	Q2	Q3	Q4	Q5	Q6	Q7	Q8	Q9	Q10
1	Anja Cuš ˇ 2021 [20]	Y	Y	Y	Y	Y	Y	Y	Y	Y	Y
2	Craig Mackie 2017 [37]	Y	Y	Y	Y	Y	Y	Y	Y	Y	Y
3	Olivia Simonsson 2021 [21]	Y	Y	Y	Y	C	Y	Y	Y	Y	Y
4	Ozlem Eylem 2021 [17]	Y	Y	Y	Y	Y	C	Y	Y	Y	Y
5	J. Kasckow 2014 [38]	Y	Y	Y	Y	Y	C	Y	Y	Y	Y
6	McManama O’Brien 2019 [39]	Y	Y	Y	Y	Y	Y	Y	Y	Y	Y
7	Tobias Schiffler 2022 [40]	Y	Y	Y	Y	Y	Y	Y	Y	Y	Y
8	J. Kasckow 2015 [41]	Y	Y	Y	Y	Y	C	Y	Y	Y	Y
9	Bethany Cliffe 2022 [24]	Y	Y	Y	Y	Y	Y	Y	Y	Y	Y
10	Natasha Josifovski 2022 [42]	Y	Y	Y	Y	C	C	Y	Y	Y	Y
11	Candice Biernesser 2021 [23]	Y	Y	Y	Y	Y	Y	Y	C	Y	Y
12	E. Baileyy 2021 [22]	Y	Y	Y	Y	C	Y	Y	Y	Y	Y
13	Rebecca Grist2018 [43]	Y	Y	Y	Y	Y	Y	Y	Y	Y	Y
14	Ana Radovic 2021 [44]	Y	Y	Y	Y	Y	Y	Y	Y	Y	Y
15	Joseph Tighe 2020 [25]	Y	Y	Y	Y	Y	C	Y	Y	Y	Y
16	Mareka Frost 2016 [45]	Y	Y	Y	Y	Y	C	Y	Y	Y	Y

“Y”: stands for yes; “C”: stands for what can’t tell; “Q”: stands for question of CASP’s items

### Meta-synthesis

**Fig. 2:**
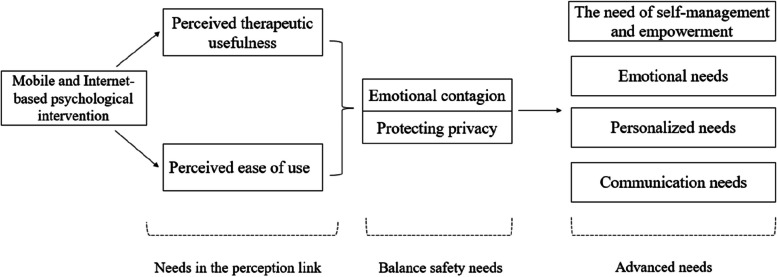
Thematic representation of research results

For a total of 149 results and 109 illustrations, the 16 qualifying investigations yielded 109 unequivocal findings (U) and 40 credible findings (C). The complete presentation of findings, illustrations, and the credibility assessment is available in Additional file 2. These results were consolidated into 14 categories and eventually distilled into three themes: needs in the perception link, balance safety needs, and advanced needs (See Additional files 3, 4, and 5 for more details). The thematic representation of research results is illustrated in Fig. 2.

#### Quality appraisal of synthesized findings

The results of the GRADE-CERQual of the Synthesized findings are shown in Additional file 6. This study summarized seven high-quality pieces of evidence, six moderate-quality pieces of evidence, and one low-quality piece of evidence.

### Synthesized finding 1: needs in the perception link

The needs in the perception link represent the initial stage and one of the most critical factors in determining whether participants use mobile Internet psychological interventions. This includes perceived ease of use and perceived therapeutic usefulness.

#### Perceived ease of use

##### Technology with accessibility (Moderate confidence)

The category of ‘technology with accessibility’ was substantiated by four studies [20, 21, 37, 42]. ​Technical usability involves maintaining the normal usage of mobile psychological intervention software, encompassing tasks such as downloading, logging on, operating, and resource acquisition. Participants identified technical accessibility and availability as two major factors that hindered their use of the software or had a negative impact on their experience (findings 42 and 49). Furthermore, some software applications' thresholds further diminished the accessibility of intervention, which is a key advantage of this mobile and internet-based psychological intervention (findings 23 and 127). Here are two examples of quotes:

*The principal reason for lack of engagement with the application was a result of technical complaints (C).*

*Potential troubles in using technology are that having an app that needs a lot of time to load and apps that take a lot of storage place on the phone (C).*

##### Suitability (Moderate confidence)

This category of suitability was supported by 12 studies [17, 20, 22–25, 37, 39, 40, 42, 43, 45]. The suitability category reflected the multidimensional nature of participants' needs for mobile and internet-based psychological intervention, encompassing how, what, who, where, and when they were used. Participants clearly expressed a preference for the use of the mobile and internet-based psychological intervention (findings 28, 88, 92, 133, and 146). The increased sense of connection and less pressure to seek assistance were the main effects of this enhanced accessibility (findings 58 and 123). Some adolescents noted that mobile and internet-based psychological intervention could facilitate their continuing care experience (findings 68 and 120). Here are three examples of quotes:

*The accessibility and convenience of using smartphones to manage NSSI was another recognized asset (U).*

*This ease, accessible and simple of use was valued by participants as they did not want to add further stress to their situation (U).*

*The majority of adolescents believed that receiving a booster of intervention content through their smartphones would be useful for their continuity of care as well as an easy mode for information retrieval (U).*

In terms of content accessed, participants expressed a desire for novel, easily accessible, specific, and feasible content related to self-injury and suicide-related behaviors through the app (findings 27, 62, 77, 84, and 101). Here is one example quote:

*Respondents were consistently eager to learn new methods of reducing tension and explicitly wanted to know better ways of dealing with this than to incur self-injury (U).*

When it came to the time and place of mobile and internet-based psychological intervention services, participants expressed a desire to be available in any state and all types of locations (findings 18, 15, 73, 81, and 146). However, application at times of crisis was crucial (findings 17, 33, 35, 73, 91, and 113). For example, when participants had intense suicidal ideation due to some triggers, they may press a button in the application and follow some simple interventions. At the same time, the software could directly send emergency notifications to specialist medical institutions for follow-up professional support. The followings are two example quotes:

*while two other participants mentioned the environment as a possible limitation, as certain skills could not be used in public places (C).*

*Provide a way for safely and easily interacting, or connecting, with others, which was particularly helpful in periods of low mood or isolation when any form of interaction was difficult (U).*

Participants in three studies indicated that mobile and internet-based psychological intervention should be integrated with specialist medical care (findings 16, 94, 136, and 139). The connection relationship ensured life safety for individuals currently experiencing severe suicidal ideation and during a period of emotional vulnerability. Participants also mentioned that the format of this online intervention was more suitable for adolescents, as they tended not to disclose their self-injury or suicidal ideation in their daily lives, opting instead to share them on their social media accounts (finding 106). The followings are two examples of quotes:

*That BlueIce would be most effective if used in conjunction with professional support, particularly for those who may be experiencing more significant distress or more severe self-harm (U).*

*Automated monitoring has the potential to detect youth who reach out for help through digital media when their comments may otherwise go unnoticed (U).*

Participants significantly recognized the need to tailor interventions for individuals with diverse cultural backgrounds (finding 59). For instance, several well-known Turkish idioms and metaphors were employed to articulate psychological distress and suicide-related behaviors in the context of mobile and internet-based psychological intervention. The cultural suitability of the online intervention contributed to Turkish immigrants feeling a greater sense of familiarity and connection. The following is an example of quote:

*All participants spoke about feeling familiar with the culturally adapted content.* They felt connected and were *also able to relate to the intervention (i.e., cultural relevance) and often found it appropriate (i.e., culturally appropriate) (U).*

#### Perceived therapeutic usefulness (High confidence)

Eleven research supported this aspect of perceived therapeutic usefulness [17, 20, 21, 23–25, 37, 40, 42, 44, 45]. On the other hand, Treatment needs were the most fundamental needs of people who had committed suicide-related behaviors and self-injury. Participants use mobile and internet-based services because their treatment needs could not be adequately satisfied in real life (findings 4, 6, 11, and 134). The primary reason given by participants for using online interventions was therapy. If participants could employ the intervention knowledge in their everyday life, it would be beneficial in lowering self-injury and suicide-related behaviors (findings 7, 14, 16, 22, 32, 48, 56, 75, 93, 99, 102, 140, 143, and 149). A proportion of participants were concerned that the online intervention would also not provide effective treatment (findings 37, 76, and 108). Interestingly, participants expressed a strong need for professional therapeutic support. They felt that online interventions needed to be deeply integrated with, rather than substituted for, professional therapeutic help, regardless of the circumstances (findings 38, 44, 95, and 132). The followings are four example quotes:

*Further negative outcomes of engaging in NSSI involve receiving invalidating reactions from their surroundings (U).*

*Observing that intervention helps them or that they feel better after using it was the most prevalent reported motivation to engage with the intervention (U).*

*Adolescents were concerned about whether a machine could effectively interpret sarcasm related to suicidal communication and did not fully trust it (U).*

*This may suggest that self-harm requires more intensive support, meaning an app may not be sufficient. Participants felt that professional support was most appropriate (U).*

#### Synthesized finding 2: balance safety needs

This theme focuses on two important contents using mobile and internet-based psychological intervention: emotional contagion and privacy protection. We needed to be careful to handle these two points in mobile psychological interventions.

#### Emotional contagion (high confidence)

Six research backed this area of emotional contagion [20, 22, 24, 37, 38, 45]. Participants of five studies reported that the constant attention and navigation to content about suicide-related behaviors and self-injury in the peer exchange community boards may have left them in a negative mood and exacerbated their illness (findings 10, 12, 20, 36, 63, 87, 97, and 117). Some online interventions used banning mention of keywords, such as suicide-related behaviors and self-injury, to avoid emotional contagion. Some participants expressed that the ban would limit their usual avenues of therapeutic expression and emotional release. Hence, participants need to balance emotional contagion with free expression (findings 119 and 145). Three typical quotes are shown below:

*They were concerned that these questions about death and suicide might make individuals relapse or even make their condition worse (C).*

*Banning discussions about suicide could be challenging for people who need to talk about their suicidality (U).*

*Safety in online services for self-injury centered around the need for moderation (U).*

#### Protecting privacy (Moderate confidence)

Nine research backed up this area of privacy protection [14, 20, 23, 25, 38, 39, 41, 42, 45]. Because mobile and internet-based psychological intervention might involve key privacy issues, participants were very concerned about privacy issues. They were very worried about how mobile and internet-based psychological intervention handled personal information and the negative consequences of privacy breaches (findings 24, 64, 72, 85, 107, 130, and 148). Participants appreciated the reliable privacy protection in several online interventions (findings 121 and 135). Adolescents, a sensitive group, mainly considered that treatment needs and privacy protection needed balance (findings 71 and 103). The followings are three examples of quotes:

*There were concerns with how the information provided might be used, particularly that it might be used by clinicians to coerce the patient to make changes or force changes on the patient (C).*

*Participants expressed concern over confidentiality and the potential consequences for an individual in responding affirmatively to questions regarding suicidal thoughts, substance use and medication non-adherence (C).*

*Most adolescents believed there should be a balance between their need for protection and for free expression and privacy (C).*

#### Synthesized finding 3: advanced need

This theme outlines the advanced needs of participants for mobile and web-based psychological interventions. It encompasses communication needs, emotional needs, personalized needs, and self-management and empowerment needs.

#### Communication needs (High confidence)

Seven research provided support for this group of communication needs [13, 20–22, 25, 39, 40]. Participants' ideas were ambivalent about the impact of mobile and internet-based psychological interventions on communication needs. Some participants felt that the therapeutic intervention should be delivered face-to-face rather than through technological products (e.g., mobile phones, software, etc.). They felt that such a format would have a negative impact on the participant's sickness and their capacity to communicate in real life (findings 25, 26, and 83). However, other participants indicated that online communication could help them to improve their communication skills and meet their need to share their experiences with people and professionals with similar experiences. Online communication could also help some participants who have barriers to communication in reality (findings 19, 90, 114, 116, 124, and 138). For example, some patients did not know how to communicate correctly with their doctor or were too embarrassed to express themselves during face-to-face interventions. They could learn the right communication skills through online interventions or express those difficult emotions through online communication. It is worth noting that, compared to others, adolescents showed a distinctly positive attitude towards online communication (findings 45 and 69). Here are three examples of quotes:

*Some interviewees also expressed concerns about the functionality of the app and the potential difficulties in providing help in an interpersonal way (U).*

*Participants often expressed the wish to talk with other people (included therapists) through future NSSI apps to learn what is most helpful (U).*

*Adolescents also reported using their phone to text or call with friends, partners, therapists, pastors, or family for support with mental health or substance use issues (U).*

#### Emotional needs (Moderate confidence)

Nine research backed up this area of emotional needs [17, 20–23, 37, 42, 44, 45]. The satisfaction of emotional needs was an essential part of being patients engaged in self-injury and suicide-related behaviors. Participants reported that the online intervention provided a non-judgmental, comfortable distance and safe emotional environment. The satisfaction of emotional needs was helpful in their treatment (findings 9, 43, 46, 54, 55, 100, 105, 110, 111, 118, 131, 141, and 144). Moreover, online interventions could help them communicate with patients going through the same experience, making them quickly gain acceptance and understanding (findings 115 and 142). The followings are two example quotes:

*Participants also found it (watching videos on their smartphones or intentionally seeking company) helpful to be in therapy where they can talk without feeling judged or receive medications (C).*

*Young people identified a general desire for understanding and a specific desire to know others had a shared experience (U).*

#### Personalized needs (High confidence)

Eleven studies supported this personalization category [14, 17, 20, 22, 24, 37–41, 45]. Personalization was a concern highlighted by almost all participants (findings 38, 40, 66, 74, 84, 89, 112, 122, and 141). Some studies explained why personalization was of such a concern to participants, mainly because of the distinct personalization of causes, experiences, and personal attitudes towards suicide-related behaviors and self-injury (findings 1, 2, 3, 5, and 96). ​The personalized content of the intervention enhances the freshness of participants and promotes ongoing engagement (findings 21, 55, 80, and 29). Two typical quotes are shown below:

*Participants commonly referred to the importance of individuality with regards to how different types of self-harm require different support and how people cope in different ways (U).*

*personalised feedback did not only motivate them to continue but it also provided a safe environment to disclose their experiences (U).*

#### The need for self-management and empowerment (High confidence)

Ten studies supported this category [17, 20, 21, 24, 25, 37, 39, 40, 43, 44]. ​Some participants indicated that a lack of motivation to improve was the main reason for their limited participation. Lack of self-management motivation results in low confidence and acceptance of online treatments (findings 13, 34, 39, 67, 78, 98, 125, and 129). Additionally, some participants also reported that the online psychological intervention helped them improve their psychological literacy, provided them with the ability to manage their own conditions, and enhanced their willingness to participate (findings 51, 57, 70, and 137). The followings are three samples:

*There were times when they are open to receiving support to stop self-harming and other times when they were less willing to accept help and to stop the act of self-harm (U).*

*Almost all participants emphasized better self-management as one of the most important benefits of the online psychological intervention (U).*

*Some participants could see the benefit of using the app as an educational tool that improved mental health literacy, thereby facilitating improved communicate*-on *with mental health professionals and self-management of diseases (U).*

## Discussion

This study provides the first qualitative synthesis of the needs of patients engaged in self-injury and suicide-related behaviors concerning mobile and internet-based psychological interventions. We conclude with three themes: needs in the perception link, balanced safety needs, and advanced needs. The synthesized findings suggest that a lack of resources, difficulties in accessing face-to-face mental health assistance, the need for additional therapy, and ease of accessibility are among the main reasons why patients engaged in self-injury and suicide-related behaviors choose mobile and internet-based psychological interventions. However, reasons for reducing patient engagement and satisfaction during online interventions included concerns about privacy protection, emotional contagion and unmet needs (such as communication needs, emotional needs, and self-management and empowerment needs).

The first theme aligns with the Technology Acceptance Model (TAM). In this theme, patients articulated their needs in the perception link of mobile and Internet-based psychological intervention. The TAM focuses on two belief constructs significantly influencing an individual’s acceptance of (intention to engage) technology: 1) perceived usefulness; and 2) perceived ease of use [46]. According to our findings, participants considered perceived therapeutic usefulness as an initial motivation for choosing an online intervention, aligning with the technology acceptance model. Patients sought assurance that the intervention was specific, individualized, varied, and sufficient to meet the basic treatment needs of the user. Therefore, health professionals should recognize the importance of enhancing the effectiveness of mobile and internet-based psychological interventions. The need for perceived ease of use suggests that patients aim to improve and broaden the convenience of mobile psychological interventions and reduce the restrictions on equipment, time, and place of use. This underscores the importance for healthcare providers to lower the threshold of use and enhance the accessibility of internet- and mobile-based psychological therapies to boost patient engagement rates in the future. For instance, professional medical and healthcare staff can collaborate in interdisciplinary teams and enlist the aid of a team specializing in digital communication technology when developing mobile and web-based psychological therapies [47]. These measures can simplify user access to mobile and Internet-based psychological help and enhance their willingness to continue using it. The results of such actions include simplified login methods, stable login, smartphone apps with lower memory consumption, and reduced device requirements (such as memory and performance) [20, 43]. Researchers should also continually strive to provide more understandable presentations, easier-to-apply intervention content, and develop more culturally appropriate content versions [17].

The results also indicated that patients who engaged in self-injury and suicide-related behaviors were more concerned about the use of mobile therapies in emergency situations than individuals with chronic diseases who were less likely to engage in such behaviors [48]. This finding shows the distinctive characteristics of patients engaged in self-injury and suicide-related behaviors, and mobile and internet-based psychological intervention should pay more attention to the protection of patients’ life safety. It is worth noting that only one of the reviewed resources focused on the cultural adaptation of different populations. Participants indicated that cultural transformation enhanced their sense of familiarity and connection to the content of the intervention. However, there was not enough research to prove the effectiveness of cultural adaptation in mobile and internet-based interventions for mental disorders [49, 50]. The difference might be related to the fact that the population of this study was the self-injury and suicidal ideation population, which differs from people with mental disorders. In the future, we need more in-depth research on high-quality cultural adaptation effectiveness in online interventions.

It is worth noting that in the second theme, the issue of emotional contagion appeared to be of particular concern to participants in this study. In contrast to some who claimed that suicide-related behaviors and self-injury are one-way contagions, several participants accepted peer-to-peer communication; therefore, learning materials on their online platforms could help them manage self-injury. Related research suggested that peer support in online interventions could provide many benefits, including a sense of community, empowerment, and access to information and support for people who engage in self-injury and exhibit suicide-related behaviors [51]. The results of this paper are consistent with the findings of related studies in which participants expressed the need for caution in dealing with the safety of self-injury and suicide-related behaviors in online communication to avoid harm to patients seeking online communication help [52]. Moreover, the risk of the negative emotional impact of online therapies might be diminished by establishing supervision teams that provide regular monitoring and training [51].

Participants expressed concerns about the security of their privacy, aligning with the findings of previous online interventions [53–55]. They considered its direct impact on trust and participation in the online intervention. However, privacy issues are inevitable in psychological interventions. In future studies, there is a need to enhance privacy protection in online interventions and devise new strategies to increase patient trust. From a technical perspective, researchers should actively seek technical support to ensure data security and user privacy when developing smart apps and online web services [56]. In addition to technical safeguards, healthcare professionals should proactively commit not to over-collect users’ personal information except for the content of psychological interventions [57]. Researchers can provide anonymous functions or actively seek third-party supervision, effectively improving users’ trust and security in an online intervention [39, 45, 58].

​In the third theme, the advanced needs of patients who engage in self-injury and suicide-related behaviors align with Maslow's hierarchy of needs theory, encompassing communication, emotion, personalization, self-management, and empowerment. Notably, personalized intervention is one of the elements emphasized by participants, which is consistent with relevant research results [59].

Participants expressed a strong desire to add customized content to the online intervention, which would help to enhance the appeal, increase user stickiness, and strengthen ongoing user engagement to promote continuity of care. In the future development of mobile and internet-based psychological interventions, personalized and newfound content should be added to meet the requirements of different types of patients. Researchers could try to express the content of psychological interventions in comics or popular online language, which are close to the daily lives of young people [20]. Besides, we can also combine online psychological intervention with games to stimulate the willingness of adolescents to persist in using. However, it is important to note that providing specific and targeted online psychological intervention programs for users or giving timely feedback and program adjustment to users’ mental states is an essential source of participants’ personalized feelings.

Moreover, in the part of emotional needs, we found the achievement of advanced needs resembles a dilemma in the real world. For instance, mobile and Internet-based psychological interventions may not adequately address the emotional needs of participants, such as contact and companionship. It remains unclear whether fulfilling these emotional needs could potentially impede the emotional development of participants in reality. Therefore, to gain a deeper understanding of the influencing factors of mobile and Internet-based psychological therapies, large-scale, multi-center research should be conducted in the future. This research should explore the satisfaction of advanced needs and the diverse impacts of mobile and Internet-based psychological interventions on real-life situations.

The advanced need for self-management may facilitate the self-improvement of participants; however, some participants also mentioned that it could potentially increase the psychological burden on patients engaging in self-injury and suicide-related behaviors. In the future, researchers can conduct qualitative research to explore the longitudinal experiences of patients dealing with suicide and self-injury through mobile psychological interventions. This research can help clarify the evolving path and influencing factors between patients' psychological stress and their ability to self-manage. To alleviate patient anxiety regarding self-management and enhance confidence in continued usage, researchers could consider implementing detailed instructions or providing encouragement and support within mobile interventions.

Mobile health has the advantages of alleviating the shortage of offline professional psychological resources, reducing patient costs, and saving waiting time [60]. However, this review found that most included studies were conducted in developed countries, such as the USA, Australia, etc., while no studies were conducted in low-and middle-income countries (LMICs). In fact, the situation of self-injury and suicide-related behaviors in LMICs is equally severe. According to statistics, three-quarters of suicides worldwide occur in LMICs, and mobile and Internet-based psychological interventions are less utilized in LMICs [61, 62].

The inadequate application of mobile and Internet-based psychological intervention in LMICs is possibly influenced by multiple real-world factors such as insufficient organization/resource availability, lack of technical experience and professionals, and limited network infrastructure and connectivity [63, 64]. To face this contradictory reality, researchers should focus on promoting the continuous application of mobile and Internet-based psychological interventions in LMICs by providing remote technical support training and personnel assistance. Reducing the dependence of online intervention on tangible resources, expanding the coverage of users, and striving to provide adequate help to people with mental illness in LMICs are also important.

### Strength and limitations

There are several strengths to this systematic qualitative review. This study is the first qualitative systematic review of the needs of patients engaged in self-injury and suicide-related behaviors based on mobile and internet-based psychological interventions, compensating for the small sample size and single perspective of individual qualitative research.

This systematic qualitative review also has some drawbacks. Firstly, despite our efforts to search as many databases as possible, we ultimately did not explore grey databases due to restrictions in resource access. Additionally, this study only included 16 papers written in English; with no publications in other languages. Moreover, eight of the incorporated documents showed some unclear risks in the CASP evaluation, potentially limiting the credibility and quality of the synthesized findings to some extent. The second point is that, despite attempts to include Chinese literature in this study, no relevant material was discovered. Consequently, only developed countries (e.g., the UK, USA, Sweden, etc.) were covered in this study, restricting the applicability of the synthesis findings to other low-income and developing nations. Regarding the quality of the 16 included studies, three studies may have recall bias in collecting qualitative data, and one study did not allow participants to review the transcribed manuscript together. Finally, the eligible articles we included did not involve the elderly, potentially limiting the interpretability of the findings in this study.

## Conclusion

The study categorized the needs of patients using mobile psychological interventions into three themes: needs in the perception link, balance safety needs, and advanced needs. The synthesis findings of this systematic qualitative review align with the applicability of the technology adaptation model and Maslow's hierarchy of needs theory in mobile and internet-based psychological interventions for patients engaged in self-injury and suicide-related behaviors. Based on the results of this study, healthcare workers can initiate by addressing the needs of patients' perception and balancing their safety needs. They can then collaborate with the communication technology team to collectively promote the application of psychological intervention products in low- and middle-income countries (LMICs). ​Researchers aim to lower the barrier for patients involved in self-harm and suicide-related behaviors to utilize mobile healthcare in the future, enhancing LMICs' acceptance and participation in mobile psychological interventions. ​Health professionals in high-income countries that have conducted mobile psychological intervention studies need to acknowledge the value and significance of higher emotional needs in mobile health and enhance the self-management skills of patients involved in self-harm and suicide-related behaviors through mobile health. This could significantly contribute to improving continuity of care standards. ​In the future, more high-quality multicenter randomized controlled trials can be conducted to explore the long-term intervention effects and other benefits of mobile psychological interventions.

### Supplementary Information

**Additional file 1.** The original search strategy for this study.

**Additional file 2. **The findings, illustrations and credibility assessment of the 16 included articles.

**Additional file 3. **One of the themes of qualitative synthesis: Needs in the perception link.

**Additional file 4. **One of the themes of qualitative synthesis:Balance safety needs.

**Additional file 5. **One of the themes of qualitative synthesis: Advanced needs.

**Additional file 6. **The results of the GRADE-CERQual of the synthesized findings.

## Data Availability

The datasets used and/or analyzed during the current study are available from the corresponding author on reasonable request.

## Abbreviations

PRISMA: Preferred Reporting Items for Systematic Reviews and Meta-Analyses statement
LMICs: Low-and middle-income countries
CASP: Critical Appraisal Skills Program
GRADE-CERQual: Confidence in the Evidence from Reviews of Qualitative research
TAM: Technology Acceptance Model
ENTREQ: Enhancing Transparency in Reporting the Synthesis of Qualitative Research statement
