# Supplementary material for: Needs for mobile and internet-based psychological intervention in patients with self-injury and suicide-related behaviors: a qualitative systematic review
Source: BMC Psychiatry. 2024 Jan 4;24:26. doi: 10.1186/s12888-023-05477-2 (PMC10768375; doi:10.1186/s12888-023-05477-2)
Supplement: Supplementary file 1 — Additional file 1. The original search strategy for this study. [file 12888_2023_5477_MOESM1_ESM.docx]

**Additional file 1: The original search strategy for this study**

Electronic databases : PubMed（Medine）, CINAHL, PsycINFO, Embase, Web of Science, Cochrane Central Register of Controlled Trials, ProQuest, CNKI, CBM ,Wan Fang, VIP.

We were searched from earliest date till April 2022 using the following subject terms and Boolean operators:

#1: (“mobile health” OR “text message” OR “E-health” OR m-health OR “mobile intervention” OR “mobile-based intervention” OR “mobile apps” OR “apps” OR computerized OR “online self-help” OR “online intervention” OR “online therapy” OR “web therapy” OR “web intervention” OR ICT)

#2: (“self-harm” OR “self-injury” OR DSH OR NSSI OR “self-burn*” OR “self-mutilation” OR “self-cutting” OR suicid*).

#3: (qualitative research OR qualitative study OR narrative research OR narrative study OR mixed research)

#1 AND #2 AND #3

The asterisks indicate wildcard operators. Database specific subject headings were also used where these map onto search terms. These additional terms are displayed in Supplementary Table 1, below.

For a comprehensive search, the databases were not restricted to study types, except for the embase database.

Supplementary Table 1 Database Specific Subject Headings Used in Literature Searches.

| Database | Subject Headings |
| --- | --- |
| **PsycInfo** | cellular phones, mobile devices, information technology, telemedicine, computer applications, computer assisted therapy, online therapy  self-mutilation; suicidal ideation; self-destructive behaviour; attempted suicide; self-injurious behaviour; suicide |

**Search results:**

**1. Web of Science (n=496)**

#1: TS=(“self-harm” OR “self-injury” OR DSH OR NSSI OR “self-burn*” OR “self-mutilation” OR “self-cutting” OR suicid*)

#2: TS=(“mobile health” OR “text message” OR “E-health” OR m-health OR “mobile intervention” OR “mobile-based intervention” OR “mobile apps” OR “apps” OR computerized OR “online self-help” OR “online intervention” OR “online therapy” OR “web therapy” OR “web intervention” OR ICT)

#1 AND #2

**2. Embase (n=39)**

#1 AND #2 AND #3

**#3**

(((**qualitative** AND **research** OR **qualitative**) AND **study** OR **narrative**) AND **research** OR **narrative**) AND **study**

**#2**

(**'self-harm'**/exp OR **'self-harm'** OR **'self-injury'**/exp OR **'self-injury'** OR **dsh** OR **nssi** OR **'self-burn*'** OR **'self-mutilation'**/exp OR **'self-mutilation'** OR **'self-cutting'**/exp OR **'self-cutting'** OR **suicid***) AND (**'mobile health'**:ab,ti OR **'text message'**:ab,ti OR **'e-health'**:ab,ti OR **'m health'**:ab,ti OR **'mobile intervention'**:ab,ti OR **'mobile-based intervention'**:ab,ti OR **'mobile apps'**:ab,ti OR **'apps'**:ab,ti OR **computerized**:ab,ti OR **'online self-help'**:ab,ti OR **'online intervention'**:ab,ti OR **'online therapy'**:ab,ti OR **'web therapy'**:ab,ti OR **'web intervention'**:ab,ti OR **ict**:ab,ti)

**#1**

**'mobile health'**/exp OR **'mobile health'** OR **'text message'**/exp OR **'text message'** OR **'e-health'**/exp OR **'e-health'** OR **'m health'** OR **'mobile intervention'** OR **'mobile-based intervention'** OR **'mobile apps'**/exp OR **'mobile apps'** OR **'apps'** OR **computerized** OR **'online self-help'** OR **'online intervention'**/exp OR **'online intervention'** OR **'online therapy'** OR **'web therapy'** OR **'web intervention'**/exp OR **'web intervention'** OR **'ict'**/exp OR **ict**

**3. PubMed(n= 610)**

("self-harm"[Title/Abstract] OR "self-injury"[Title/Abstract] OR "DSH"[Title/Abstract] OR "NSSI"[Title/Abstract] OR "self burn*"[Title/Abstract] OR "self-mutilation"[Title/Abstract] OR "self-cutting"[Title/Abstract] OR "suicid*"[Title/Abstract]) AND ("mobile health"[Title/Abstract] OR "text message"[Title/Abstract] OR "E-health"[Title/Abstract] OR "m-health"[Title/Abstract] OR "mobile intervention"[Title/Abstract] OR "mobile-based intervention"[Title/Abstract] OR "mobile apps"[Title/Abstract] OR "apps"[Title/Abstract] OR "computerized"[Title/Abstract] OR "online self-help"[Title/Abstract] OR "online intervention"[Title/Abstract] OR "online therapy"[Title/Abstract] OR "web therapy"[Title/Abstract] OR "web intervention"[Title/Abstract] OR "ICT"[Title/Abstract])

**4. Cochrane library(n=347)**

#1: TS=(“self-harm” OR “self-injury” OR DSH OR NSSI OR “self-burn*” OR “self-mutilation” OR “self-cutting” OR suicid*)

#2: TS=(“mobile health” OR “text message” OR “E-health” OR m-health OR “mobile intervention” OR “mobile-based intervention” OR “mobile apps” OR “apps” OR computerized OR “online self-help” OR “online intervention” OR “online therapy” OR “web therapy” OR “web intervention” OR ICT)

#1 AND #2

**5. CINHAL(n=206)**

#1: TS=(“self-harm” OR “self-injury” OR DSH OR NSSI OR “self-burn*” OR “self-mutilation” OR “self-cutting” OR suicid*)

#2: TS=(“mobile health” OR “text message” OR “E-health” OR m-health OR “mobile intervention” OR “mobile-based intervention” OR “mobile apps” OR “apps” OR computerized OR “online self-help” OR “online intervention” OR “online therapy” OR “web therapy” OR “web intervention” OR ICT)

#1 AND #2

**6. Proquest(n=73)**

#1: TS=(“self-harm” OR “self-injury” OR DSH OR NSSI OR “self-burn*” OR “self-mutilation” OR “self-cutting” OR suicid*)

#2: TS=(“mobile health” OR “text message” OR “E-health” OR m-health OR “mobile intervention” OR “mobile-based intervention” OR “mobile apps” OR “apps” OR computerized OR “online self-help” OR “online intervention” OR “online therapy” OR “web therapy” OR “web intervention” OR ICT)

#1 AND #2

**7.PsyINFO(n=106)**

#1: TS=(“self-harm” OR “self-injury” OR DSH OR NSSI OR “self-burn*” OR “self-mutilation” OR “self-cutting” OR suicid*)

#2: TS=(“mobile health” OR “text message” OR “E-health” OR m-health OR “mobile intervention” OR “mobile-based intervention” OR “mobile apps” OR “apps” OR computerized OR “online self-help” OR “online intervention” OR “online therapy” OR “web therapy” OR “web intervention” OR ICT)

#1 AND #2

**8.CNKI(n=0)**

#1: TS=(“zi shang” OR “zi wo shang hai” OR “fei zi sha xing zi shang” OR “zi can” OR“zi sha”)

#2: TS=(“yi dong jian kang” OR “duan xin” OR “dian zi jian kang” OR “yi dong gan yu” OR “shou ji apps” OR “apps” OR dian nao OR “zai xian zi wo gan yu” OR “zai xian gan yu” OR “zai xian zhi liao” OR “wang luo zhi liao” OR tong xin ji shu)

#1 AND #2

**9.CBM(n=0)**

#1: TS=(“zi shang” OR “zi wo shang hai” OR “fei zi sha xing zi shang” OR “zi can” OR“zi sha”)

#2: TS=(“yi dong jian kang” OR “duan xin” OR “dian zi jian kang” OR “yi dong gan yu” OR “shou ji apps” OR “apps” OR dian nao OR “zai xian zi wo gan yu” OR “zai xian gan yu” OR “zai xian zhi liao” OR “wang luo zhi liao” OR tong xin ji shu)

#1 AND #2

**10.WANFANG(n=0)**

#1: TS=(“zi shang” OR “zi wo shang hai” OR “fei zi sha xing zi shang” OR “zi can” OR“zi sha”)

#2: TS=(“yi dong jian kang” OR “duan xin” OR “dian zi jian kang” OR “yi dong gan yu” OR “shou ji apps” OR “apps” OR dian nao OR “zai xian zi wo gan yu” OR “zai xian gan yu” OR “zai xian zhi liao” OR “wang luo zhi liao” OR tong xin ji shu)

#1 AND #2

**11.VIP(n=0)**

#1: TS=(“zi shang” OR “zi wo shang hai” OR “fei zi sha xing zi shang” OR “zi can” OR“zi sha”)

#2: TS=(“yi dong jian kang” OR “duan xin” OR “dian zi jian kang” OR “yi dong gan yu” OR “shou ji apps” OR “apps” OR dian nao OR “zai xian zi wo gan yu” OR “zai xian gan yu” OR “zai xian zhi liao” OR “wang luo zhi liao” OR tong xin ji shu)

#1 AND #2
