# Supplementary material for: Needs for mobile and internet-based psychological intervention in patients with self-injury and suicide-related behaviors: a qualitative systematic review
Source: BMC Psychiatry. 2024 Jan 4;24:26. doi: 10.1186/s12888-023-05477-2 (PMC10768375; doi:10.1186/s12888-023-05477-2)
Supplement: Supplementary file 2 — Additional file 2. The findings, illustrations and credibility assessment of the 16 included articles. [file 12888_2023_5477_MOESM2_ESM.docx]

**Additional file 2: The findings, illustrations and credibility assessment of the 16 included articles**

1. **“U” is meaning:** Unequivocal (findings accompanied by an illustration that is beyond reasonable doubt and; therefore, not open to challenge)
2. **“C” is meaning:** Credible (findings accompanied by an illustration lacking clear association with it and therefore open to challenge)
3. Unsupported (findings are not supported by the data)

| Study ID: Anja Cuš ˇ 2021 | |
| --- | --- |
| Finding **1** | NSSI had self-punishing qualities. **(U)** |
| illustration | P14: “Ok. So, it mostly arises from the situation that triggered that, are the situations where I say: ‘Yes, you are a disappointment’, so I say it to myself, that I am a disappointment and need to be punished for that, so ‘for this mistake you need to be punished’ and I accept that. And well, that is how it is and then I did it.” |
| Finding2 | Participants’ accounts of NSSI were often ambivalent. **(C)** |
| illustration | summary by author |
| Finding3 | a few of them experienced feelings of guilt after engaging in NSSI. **(C)** |
| illustration | summary by author |
| Finding4 | Further negative outcomes of engaging in NSSI involved receiving invalidating reactions from their surroundings**(U)** |
| illustration | P10: “I mean obviously, like covering scars and like when people see scars like they get freaked out and they start asking questions, so that’s obviously something that I try to avoid.” |
| Finding5 | one participant described it as a routine and another one as a part of her life. **(U)** |
| illustration | P13: “For me it simply became a part of my life/ . . . /. It is also difficult to stop with it, as it is so much inside of my everyday life.” |
| Finding6 | most of them said they struggle with reaching out for help**(U)** |
| illustration | I: “And what do you need, when you have the feeling you want to injure yourself?”P5:“Simply to talk, but I have the problem for example, that I cannot say, yes, ‘Hi, I needhelp, I need someone to talk to.’” |
| Finding7 | Coping with NSSI urges and thoughts by successfully implementing (mostly dialectical behavioural therapy—DBT) skills. **(U)** |
| illustration | P11: “I do the opposite. I learned that in [the DBT] skills group. I do the opposite of the feeling that I have. For example, if I am sad and feel like crying, I wipe my tears and put a smile on my face.” I: “How was that before the skills group?” P11: “Then I would cut myself.” |
| Finding8 | Some participants were successful in resisting NSSI through thinking that this behaviour would hurt people around them. **(C)** |
| illustration | summary by author |
| Finding9 | Participants also found it (watching videos on their smartphones or intentionally seeking company) helpful to be in therapy where they can talk without feeling judged or receive medications. **(C)** |
| illustration | summary by author |
| Finding10 | Listening to music was helpful as well but they noted that it can also lead to a negative affective outcome. **(C)** |
| illustration | summary by author |
| Finding11 | Reaching out was not always productive. **(U)** |
| illustration | P9: “I called a hotline and that did not go well/ . . . /And then she repeated all the time that life is beautiful. And then I hang up.” I: “Ok. And when you called, what would help you more than someone who says that life is beautiful?” P9: “[To hear] that life can be absolutely crap.” |
| Finding12 | Using the Internet in a counterproductive manner through exchanging tips on how to self-injure. **(C)** |
| illustration | summary by author |
| Finding13 | Some of them never tried to prevent NSSI or described NSSI as too fast to find a solution. **(C)** |
| illustration | summary by author |
| Finding14 | their well-developed coping strategies were sometimes not available or despite being available, they nevertheless engaged in NSSI. **(U)** |
| illustration | P10: “So, I mean, I think letting out like your anger in art room doesn’t really work because it’s not as satisfying as like cutting or whatever; but the whole thing is that you need to know that it’s bad for you and that it like it hurts people around you and I mean, yeah, that is my answer.” |
| Finding15 | Coping strategies changed over time and were also facilitated or hindered by environment. **(U)** |
| illustration | I: Do you try to distract yourself from self-injuring? P3: Often. I: How do you do this? P3: It depends on where I am./ . . . /At home drawing, music, series or a book. In school, nothing works for me anyways. |
| Finding16 | they felt that interventions should offer a complete distraction or help in reaching different thoughts. **(U)** |
| illustration | P9: “So, with me it is so, I cannot think clearly in the situation, to solve problems is then not even an option, it is the feeling of not being able to do anything, that leads to it.” |
| Finding17 | Support that they wish to receive needs to be specific for NSSI and that includes support during an acute state of mind. **(U)** |
| illustration | P14:“If there would be a button that can do it—to help immediately. That would be forme a total rescue./. . ./It also depends on umm the degree, how severely one is affected byself-injury. There are some, who can resist it and some who really cannot resist it./. . ./And that one would before it comes that far, that one would get an immediate help.” |
| Finding18 | they want to feel supported through an app at diverse time points, e.g., before NSSI, after an NSSI act and even after they in general stop engaging in NSSI. **(C)** |
| illustration | summary by author |
| Finding19 | Participants often expressed the wish to talk with other people (included therapists) through future NSSI apps to learn what is most helpful. **(U)** |
| illustration | P2: “Well, perhaps so that you can get into contact with others, can somehow talk, because there often are unfamiliar people, with unfamiliar people it is often easier to talk compared to those that know you, those with whom you are together the whole day. Perhaps like a chat, so that a lot of people can write with each other, like a group.” P2: “Well, adults that have a clue about it, that how I . . .” I: “So, professionals.” P2: “Yes, rather professionals.” |
| Finding20 | these exchanges should be moderated because talking with peers can also trigger NSSI. **(U)** |
| illustration | I: “So not peers, but. . .” P2: “No, not that, because that can go wrong, you never know.” |
| Finding21 | the wish of young people to personalize interventions and make them more relatable to their experience. **(U)** |
| illustration | P11: “What would I insert into it? That you first know when you the whole time onlyfeel so so sad, that you know why are you sad. /. . ./I would insert something about meinside. For example, what is my name and how I live. No clue, I do not know, but firsttell a bit about myself.” |
| Finding22 | Observing that intervention helps them or that they feel better after using it, was the most prevalent reported motivation to engage with the intervention. **(U)** |
| illustration | I: “Which rewards should an app have that it keeps you motivated to use it?” P8: “Only that it helps, that is actually the only thing.” |
| Finding23 | Potential troubles in using technology is that having an app that needs a lot of time to load and apps that take a lot of storage place on the phone. **(C)** |
| illustration | summary by author |
| Finding24 | Participants expressed data management and security concerns. **(C)** |
| illustration | summary by author |
| Finding25 | in apps there is no one on the other side who would actively listen to you. **(U)** |
| illustration | P14: “So, I know many apps, I don’t know, how my app would look like, but from what I’ve seen, there are these AI, these fake chats, where one can write into and one can talk with a robot, so that one does not feel alone or something like that. It can be good for some people, but for the others, it can be quite disappointing to notice, that one does not talk with a person. So, it has a different feeling to it. One knows exactly what is human and what is not human.” |
| Finding26 | people should talk with other people instead of using technology to manage NSSI. **(U)** |
| illustration | I: “How do you use the Internet to feel better?” P15: “/ . . . /At the moment not at all, because I prefer to talk with people who I know, to talk about it in person. Mostly I then meet my friends and talk with them.” |
| Finding27 | An attractive aspect of using apps to manage NSSI is novelty and participants’ wish to regularly see new content. **(C)** |
| illustration | summary by author |
| Finding28 | Accessibility and convenience of using smartphones to manage NSSI was another recognized asset. **(U)** |
| illustration | P10: “Yeah, I mean, your phone, definitely I mean, I guess everyone has like their music on their phone and like games or different chats or like social media, so, yeah, so I think that’s something that is like since it’s always like attached to you, you can use it to actually benefit you in that way.” |
| Finding29 | use of gamification elements. **(U)** |
| illustration | P1: “It could be like a game. So that when you . . . Or one should take a photo of their hand if they injured themselves or not. And you log that in. Like a photo album. Yes. Like a photo album where you take a photo of your hand every day whether you injured yourself or not. And then at the end of the month you receive an award or so. Or, I don’t know, a voucher or something like that.” |
| Finding30 | Participants were interested in using formats that are visually appealing, have a clear structure, are simplified, easy to use and are logically divided into categories. **(C)** |
| illustration | summary by author |
| Finding31 | expressed openness towards using apps for NSSI. **(U)** |
| illustration | P2: “I cannot imagine it [how it would look like]. I found the idea interesting, that is why I do this [the interview]. It would be cool if there is something like that.” |
| Finding32 | the interventions to be meaningfully implemented they will not only need to fit into the daily life of young people but will also need to deliver interventions that support adaptive coping strategies. **(C)** |
| illustration | summary by author |
| Finding33 | the topics of helpfulness and support in crisis moments seem especially important to young people with mental health problems. **(C)** |
| illustration | summary by author |
| Study ID: Craig Mackie 2017 | |
| Finding34 | Trust in the functioning and effectiveness of the application was associated with overall participant engagement. **(U)** |
| illustration | Interviewer: “Was the therapy sufficient to meet your emotional needs?” Participant: “It was. I did feel supported. It was sufficient.” Interviewer: “During the times that you were away, not directly in the face-to-face therapy, there was the BEACON button, which... potentially would be sort of like an emergency contact. Did that give you any feeling of security? Or was it, because of the bugs, kind of a non-issue?” Participant: “It was a non-issue. It didn’t give me any security because it didn’t work.” (P4) |
| Finding35 | Participants were concerned about their ability and need to use phone applications in times of crisis. **(U)** |
| illustration | Participant: “The BEACON function itself was enough to give me a nudge to get my safety plan going. Because my problem coming into this was that I pretty much lost situational awareness and won’t realize that I’m getting into such a mental state. But the BEACON button was able to overcome that.” Interviewer: Participant: “Well it certainly was dual purpose, ‘A’ it reminded me that there was help out there, and ‘B’ It, for me personally, it directed me towards my safety plan.” (P2) |
| Finding36 | The BEACON button also elicited a certain amount of anxiety in some participants. **(U)** |
| illustration | “And even having the BEACON button right at the bottom of the screen, really massive and big, it’s good to have it there for emergencies, but I was kind of nervous that I would tap it (laughs).” (P3) |
| Finding37 | some concern expressed that the supports underlying the BEACON button would be insufficiently resourced. **(U)** |
| illustration | “[My favorite thing about the app] was probably the button to contact [support during] an emergency. Because there are different circumstances where you [might reach out], like, it’s not a 9-1-1 situation, but it’s also not, like, a wait six weeks for an appointment situation. Then again, I don’t know if there are the resources for people to be able to access a psychiatrist, ah, just by pressing a button (laughing).” (P1) |
| Finding38 | hope for greater customisation of the feature, integration with a crisis line or extra monitoring by the therapist. **(C)** |
| illustration | summary by author |
| Finding39 | trust in the application’s effectiveness and function, and trust in the therapeutic alliance, appeared to influence participant feelings of connectedness. **(C)** |
| illustration | summary by author |
| Finding40 | a desire for more personalised features was consistently expressed. **(U)** |
| illustration | Participant: “just like, interesting, encouraging things...” Interviewer: “They had the Daily Thoughts, did you have that active?” Participant: “Yeah, but even making it more personal... [drawing on] values that are currently in the app that the user is using, [so that] the app feels like it’s helping them personally. If it’s just those general thoughts or those general motivations, people can get to the point where they just ignore them... But if it’s something that’s interesting and specifically geared to them.(P3) |
| Finding41 | higher levels of mental health literacy acting as deterrent to engagement. **(U)** |
| illustration | “Was more just to keep [the primary investigator] in the loop than useful for me... I have all these different apps that maybe [already] do one of the things. Like I have a calendar app, I have a note writing app... I like that you were trying to put them all in one place, and that the emergency stuff is there and what not, but any way to make it more smooth...” (P3) |
| Finding42 | the principal reason for lack of engagement with the application was a result of technical complaints. **(C)** |
| illustration | summary by author |
| Finding43 | they felt sufficiently emotionally supported during the course of the therapeutic relationship in study procedure. **(C)** |
| illustration | summary by author |
| Study ID: Olivia Simonsson 2021 | |
| Finding44 | Support From the Therapist Despite Distance. **(U)** |
| illustration | You could just ask anything, also questions that I thought were stupid, but still. Otherwise [without the therapist] it would have felt lonelier, as if you were just doing it by yourself, like nobodycared. Now there was someone who was there that wrote to you, after all. You got the response quickly when you sent a message, it felt good. [Adolescent #5] |
| Finding45 | Adolescents appreciated the online communication with the therapist, and many preferred it to talking face-to-face. **(C)** |
| illustration | summary by author |
| Finding46 | the physical distance to the therapist seemed to increase their willingness to share sensitive personal information. **(U)** |
| illustration | simply find it easier to write, to get more time to think about exactly how to formulate myself...it can be easier to say things I don’ t like to say aloud. [Adolescent #5] |
| Finding47 | The fictional characters in the modules were perceived as relatable and created a sense of normalization. **(U)** |
| illustration | There were always examples of four people, and I could always recognize myself in at least one of them. Sometimes you might not recognize yourself up to a hundred percent, but there was always something you could recognize that made you feel less alone and “all right, it’s not just me who has this problem.” [Adolescent #4] |
| Finding48 | Adolescents appreciated it. Practicing skills in the mobile app and getting suggestions of what to do made the treatment more present and supported everyday skills training. **(U)** |
| illustration | I think the app was the best. I probably logged in to it more times than I really needed. More as a reminder to me.... Sometimes it was hard to remember what to do in a situation when I was feeling very, very bad. If I logged in to the app, I had more control. Otherwise, I find it very difficult to come up with it [strategies] myself. [Adolescent #9] |
| Finding49 | The reasons mentioned for not using the mobile app were not experiencing the need or technical issue. **(C)** |
| illustration | summary by author |
| Finding50 | self-responsibility can be empowering as well as distressing. **(C)** |
| illustration | summary by author |
| Finding51 | Other positive aspects of self-responsibility were connected to empowerment. **(U)** |
| illustration | t didn’t feel like I was troubling anyone else in any way with my mental health problems. It was just me trying to get better. [Adolescent #3] |
| Finding52 | When adolescents felt that they did not meet expectations, some ended up procrastinating and avoiding treatment. **(U)** |
| illustration | When I felt that I would not be able to do as many homework assignments as I wanted to do, then I did nothing instead, and finally I felt more str essed because I did nothing.... [Adolescent #3] |
| Finding53 | Rarely did adolescents or caregivers mention such concerns（ insecurities about what was right and enough ） to their therapists. **(U)** |
| illustration | One problem was that I had a hard time formulating answers to the questions in the module, so I don’t really know if I...came through with all my thoughts to the therapist. I thought the messages worked well, but I was always unsure how much I should write in the questions in modules-How deep should I go? [Adolescent #1] |
| Finding54 | the improvements expressed by adolescents were increased emotional awareness and acceptance, courage to be who you are, skillful communication of emotions, and reaching out for help before making the situation worse. **(U)** |
| illustration | I have become very much more aware of how I really work and how emotions and thoughts work and...I don’t know , just a lot of knowledge. It’s been amazing! And can help friends a little bit too, so it’s cool. [Adolescent #1] |
| Study ID： Ozlem Eylem 2021 | |
| Finding55 | personalised feedback did not only motivate them to continue but it also provided a safe environment to disclose their experiences. **(U)** |
| illustration | Receiving feedback was like exchanging letters with someone.... Sometimes you cannot talk to everyone about certain things. But receiving feedback and being able to respond to it, was like a relief.... As I went through them, I kept on discovering new things about myself Participant A. |
| Finding56 | The psycho-educational aspect of the ex-excises and the feedback helped them to understand these crisis situations. **(U)** |
| illustration | I was going through a trauma....and was not able to make much sense of what was happening to me... the feedback helped me to make sense of it all. It helped me to explain things from a scientific point of view Participant D. |
| Finding57 | Almost all participants emphasised better self-management as one of the most important benefits of the intervention. **(U)** |
| illustration | It [following the intervention] gave me some peace of mind as I was doing something at least Participant H. |
| Finding58 | Suitability of the intervention was emphasised as a strong facilitator for feeling connected. **(U)** |
| illustration | I felt the exercises were suitable with my life style...Working with a coach was also helpful in terms of feeling connected...I feel I gained skills that I could use for the rest of my life Participant A. |
| Finding59 | All participants spoke about feeling familiar with the culturally adapted content. They feel connected were also able to relate to the intervention (i.e., cultural relevance) and often found it appropriate (i.e.,culturally appropriate). **(U)** |
| illustration | I think all the examples were appropriate to the Turkish culture...They were also representative of the types of problems that migrant populations are likely to face Participant G. |
| Finding60 | the ‘self-help’ principles made it difficult to use the intervention. **(U)** |
| illustration | The difficulty with the online therapy is that, we need to do things on our own. When you see a psychologist.... when there is a person in front of you.... you feel more in control... after all not feeling in control is the main rea-son why we need psychological help.... Isn’t it? Participant H. |
| Finding61 | Not feel connected for participant as having severe suicidal ideation. **(U)** |
| illustration | The intervention was for severe cases [people who have intense thoughts about suicide] .... I am not in that group.... so sometimes the questions and the exercises were not so relevant to me. I asked myself if this is how they really think about me. Am I considered as a “nut case”? This was affecting my willingness to participate.... You know...how you feel changes your decisions.... Participant H. |
| Finding62 | More directive approach. (U) |
| illustration | There were many exercises....and I needed to find out which one works better for me.... I didn’t quite catch that in the beginning...It worried me...I felt I was not in control.... I think there could be more personalised guidance so that it’s easier to find the right exercises Participant I. |
| Study ID: J. Kasckow 2014 | |
| Finding63 | They were concerned that these questions about death and suicide might make individuals relapse or even make their condition worse. **(C)** |
| illustration | summary by author |
| Finding64 | There were concerns with how the information provided might be used, particularly that it might be used by clinicians to coerce the patient to make changes or force changes on the patient. **(C)** |
| illustration | summary by author |
| Finding65 | Individuals answering these questions may not answer honestly. **(C)** |
| illustration | summary by author |
| Finding66 | participants requested that certain ques-tions exhibited greater specificity. **(U)** |
| illustration | with the following question—‘‘How do you see the future?’’ participants commented that a specific time frame was needed. |
| Study ID: McManama O’Brien 2019 | |
| Finding67 | Acceptability of the section on interest and confidence in changing appeared to depend on level of confidence. **(C)** |
| illustration | summary by author |
| Finding68 | The majority of adolescents believed that receiving a booster of intervention content through their smartphones would be useful for their continuity of care as well as an easy mode for information retrieval. **(U)** |
| illustration | “Definitely, my number one use of communication, regardless of any sort of emergency or anything, is my phone.” |
| Finding69 | Adolescents also reported using their phone to text or call with friends, partners, therapists, pastors, or family for support with mental health or substance use issues. (U) |
| illustration | used their phone to text or call with friends, a partner for support, or family for support. (Participant 5) |
| Finding70 | Adolescents also wanted the goals they identified during the in-person intervention to be incorporated into the app. **(C)** |
| illustration | summary by author |
| Finding71 | adolescents advocated for the inclusion of a method for interacting with peers anonymously for support. **(U)** |
| illustration | “So a reminder of—that you’re not alone and—Can see—or not see other people who are trying to do the same thing, but find a way to give each other motivation or something.” |
| Finding72 | In general, adolescent participants were not interested in GPS tracking and location-based messaging (i.e., when they approach high-risk locations), with one participant calling it “creepy.” **(U）** |
| illustration | could potentially be helpful, but would need to have the option to turn that off. (Participant 1) |
| Finding73 | One adolescent highlighted the importance of receiving this booster in the vulnerable time period immediately following discharge. **(U)** |
| illustration | “Especially just getting out of the hospital, people are a little bit—I don’t wanna say culture shock, because they weren’t out for too long. It’s the same culture, it’s just you were out of society and then got thrown back in. You’re just having some sort of check-up. A nice little check-up.” |
| Finding74 | Adolescents emphasized the need for personalization with respect to goals and motivators. **(C)** |
| illustration | summary by author |
| Study ID: Tobias Schiffler 2022 | |
| Finding75 | These participants reported various ways of seeking relevant information about DBT skills, mainly through online platforms and self-help forums. **(C)** |
| illustration | summary by author |
| Finding76 | most of these participants conveyed their frustration that many suggestions for self-help were not inspiring enough or unhelpful in dealing with their symptoms. **(C)** |
| illustration | summary by author |
| Finding77 | Respondents were consistently eager to learn new methods of reducing tension and explicitly wanted to know better ways of dealing with this than to incur self-injury. **(U)** |
| illustration | I don’t feel like I already know enough skills and I should seriously work on that now, but I am hindered by the distraction, a certain indifference that sometimes appears. (Female, Age 21) |
| Finding78 | In terms of intrapersonal risk factors to have led to self-injury, poor self-worth and self-hatred were recurring themes. **(C)** |
| illustration | summary by author |
| Finding79 | the use of mobile devices in general, as well as certain apps as effective tools to combat NSSI and suicidal behavior. **(C)** |
| illustration | summary by author |
| Finding80 | Some respondents stated that suggestions from healthcare professionals regarding general skills were often insufficient, as skills could only be effective if they were personalized. **(C)** |
| illustration | summary by author |
| Finding81 | while two other participants mentioned the environment as a possible limitation, as certain skills could not be used in public places. **(C)** |
| illustration | summary by author |
| Finding82 | In general, respondents had positive opinions about the use of mobile apps in terms of emotion regulation. **(U)** |
| illustration | I do believe that in some cases an app can help really well with tension, but I actually believe that it is difficult to program an app in such a way or to make it so user-friendly in a way that when you are in a high level of tension that you can actually experience the app as helpful. (Female, Age 21) |
| Finding83 | some interviewees also expressed concerns about the functionality of the app and the potential difficulties in providing help in an interpersonal way. **(U)** |
| illustration | there can certainly be situations where an app is sufficient. But I think that the interpersonal aspect gets lost. For me, one of my best skills is talking to someone. (Female, Age 21) |
| Finding84 | respondent suggested integrating an option to adapt the provided content based on individual experience. **(C)** |
| illustration | summary by author |
| Study ID: J. Kasckow 2015 | |
| Finding85 | Participants expressed concern over confidentiality and the potential consequences for an individual in responding affirmatively to questions regarding suicidal thoughts, substance use and medication non-adherence. **(C)** |
| illustration | summary by author |
| Finding86 | participants requested that certain questions exhibit greater specificity. **(U)** |
| illustration | ‘‘Have you been around people who have been abusing drugs or alcohol?’’, participants commented that a specific time frame needed to be specified. |
| Study ID: Bethany Cliffe 2022 | |
| Finding87 | constantly monitoring mood as low may have a detrimental effect on well-being. **(C)** |
| illustration | summary by author |
| Finding88 | Participants liked BlueIce’s simplicity and ease of use and felt that it could provide comfort and support. **(U)** |
| illustration | “calm you down and almost realise that things aren’t as stressful” [p13]. |
| Finding89 | The personalizable options were also well received as participants felt this would make the app widely applicable. **(U)** |
| illustration | that’s a very clear toolbox of things that you can do and you can adapt to suit yourself, which is incredible[p02]. |
| Finding90 | so including a chat feature could be beneficial—particularly a peer support chatroom or a webchat with a mental health professional. **(C)** |
| illustration | summary by author |
| Finding91 | Most of the functions identified related to support that could be provided in crisis moments. **(U)** |
| illustration | “Disengage yourself from those thought” [p06]. |
| Finding92 | Many participants felt that the immediate accessibility of BlueIce addressed limitations of other forms of support. due to simplicity, increased ease, and immediacy of access wherever you are as. **(U)** |
| illustration | “Everyone has a phone nowadays, and has their phone in front of them” [p06]. |
| Finding93 | how BlueIce may be able to offer the same level of support but without the added burden of disclosing self-harm before they feel able or ready to. **(U)** |
| illustration | Some people don’t want to talk to people, so some people don’t want to seek the help from counselling but to be able to have this accessible to them might help them in ways that counselling would but without the counselling. [p04] |
| Finding94 | that BlueIce would be most effective if used in conjunction with professional support, particularly for those who may be experiencing more significant distress or more severe self-harm. **(U)** |
| illustration | I think it’s something that the university counselling services should offer, but I think also, yeah, you’ve obviously got people who might be experiencing more milder low moods ... but maybe if they use this app and find that they’re just feeling low all the time then that would be the time to kind of, take it a bit further with the university. [p01] |
| Finding95 | This may suggest that self-harm requires more intensive support, meaning an app may not be sufficient. participants felt that professional support was most appropriate. **(U)** |
| illustration | “I needed someone to talk to, I didn’t need an app” [p10] |
| Finding96 | Participants commonly referred to the importance of individuality with regards to how different types of self-harm require different support and how people cope in different ways. **(U)** |
| illustration | “It might work for me, but I know other people who would rather have face-to-face [support] or have someone there, but I’d rather not have somewhere there, but yeah that’s just me.” [p26] |
| Finding97 | there was also some concern around whether seeing lots of consecutive “bad days” would discourage the user: **(U)** |
| illustration | “you may see sort lots of really negative days and get really bogged down in that and think ‘why bother’”. [p11] |
| Finding98 | Potential barrier included the perceived effort involved in using the app and the motivation that would be required to engage with it. **(U)** |
| illustration | It depends on your kind of mind state at the time and maybe how bad it is or generally what you’re like as a person, but I’m not sure if I was feeling really, really crap and I had an urge to self-harm, I’m not sure I would go through my phone and scroll through an app. [p14] |
| Study ID: Natasha Josifovski 2022 | |
| Finding99 | the text messages served as a reminder for the participant to focus on their mental health and included useful tips to improve their mental state. **(U)** |
| illustration | “a good reminder of what you needed to do and a few good resources” |
| Finding100 | the text messages were often a comfort in knowing that someone was there and that someone cares. **(U)** |
| illustration | “Make [me] feel less alone. I felt alone, but when I saw the text message I thought oh there’s someone there” |
| Finding101 | the relative ease of access of the text messages and their accompanying content. **(U)** |
| illustration | “I could always come back to them, they were right there in my text messages.” |
| Finding102 | the messages could have the potential to serve as a triggering reminder. **(U)** |
| illustration | “Might remind you that you’re at a bad place” |
| Study ID：Candice Biernesser 2021 | |
| Finding103 | Most adolescents believed there should be a balance between their need for protection and for free expression and privacy. **(C)** |
| illustration | summary by author |
| Finding104 | They felt that monitoring these private conversations would diminish their ability to be themselves and limit their opportunities for peer support. **(C)** |
| illustration | summary by author |
| Finding105 | Some youth were concerned about burdening their therapist with issues that felt inconsequential，They described apprehension in initiating these conversations and wanted prompting from therapists. **(U)** |
| illustration | I think if it bothered me a lot, I wouldn’t be able to tell him...at all. Like I would have to be asked a question pertaining to it. I probably wouldn’t talk about it out of the blue. |
| Finding106 | automated monitoring has the potential to detect youth who reach out for help through digital media when their comments may otherwise go unnoticed. **(U)** |
| illustration | I’m sure that a lot of kids turn to social media, because they don’t know how to turn to the people in real life. And sometimes it’s easier hiding behind a screen. [adolescent] |
| Finding107 | their primary concern is the loss of privacy associated with releasing DMU for automated monitoring. **(U)** |
| illustration | You know, I guess with social media I would be a little more comfortable just because it’s...already out there anyway. I think I [feel] more adversely at the text side. [parent] |
| Finding108 | Adolescents were concerned about whether a machine could effectively interpret sarcasm pertaining to suicidal communication and not fully trust it. **(U)** |
| illustration | Some people are serious, some people are just joking, some people are suicidal and joking. But there are so many jokes about wanting to kill yourself, that it would be too hard to actually pinpoint the actual people who are at risk. [adolescent] |
| Finding109 | They suspected that some may change their behavior to negate the potential for risk alerts to be generated. **(U)** |
| illustration | It would make people go off of it. They’d find their way around it. Or it’d be completely fake people trying to be happy so that they wouldn’t get monitored. But, at the same time, no monitoring is also kind of an issue. [adolescent] |
| Study ID: E. Baileyy 2021 | |
| Finding110 | participants experiencing Affinity as a positive environment, wherein they felt both safe and supported. **(U)** |
| illustration | “I think it was just that knowledge that it was a safe space” |
| Finding111 | This sense of safety was discussed both in terms of being shielded from negative content and feeling safe from ridicule or judgment. **(U)** |
| illustration | “Most of the time [supporting others] felt kind of nice. It can also be frustrating when people don't want to listen” |
| Finding112 | Many valued when moderators reached out with personalized messages. **(U)** |
| illustration | [The moderators] sent us really big paragraphs which I really liked. I don't care how long they are, but the fact that they took time to write that and it was meaningful, it was really supportive. [ID 16, aged 16 years] |
| Finding113 | provide a way for safely and easily interacting, or connecting, with others, which was particularly helpful in periods of low mood or isolation when any form of interaction was difficult. **(U)** |
| illustration | Previous, what I would normally do is just cut contact off completely. This gave me one thing that I could stay on. [ID 12, aged 23 years] |
| Finding114 | Many participants specifically spoke about valuing participation in a network of other people with similar lived experiences. **(U)** |
| illustration | You read posts and it would be something, you'd be like oh I've had that thought or that's how I feel. Then in your head you're like oh, this person's feeling that way too. [...] You read that post and think oh okay I'm not crazy, I'm not the only person that thinks that. [ID 08, aged 22 years] |
| Finding115 | how this led to them feeling particularly validated and understood by the other users. **(U)** |
| illustration | That was something I could talk about on Affinity, and be candid about on Affinity and not - I could tell my friends, but there was - it was - there was something about saying it in front of other people who understood about this. [ID 11, aged 18 years] |
| Finding116 | they were able to learn from other users. **(U)** |
| illustration | “More useful than people that haven't been through this kind of thing, purely because they themselves know more or less what helps and what doesn't” (ID 05, aged 22 years). |
| Finding117 | A total of 2 subthemes related to the source of this anxiety were identified: fear of negative evaluation and fear of causing harm. **(U)** |
| illustration | “The first one to comment,” for fear that other users “won’t react to it and you'd just be sitting there and no-one will give a shit about what you said.” |
| Finding118 | a lack of timely or prompt acknowledgment or validation from the network in relation to comments or disclosures made on Affinity had a negative impact. **(U)** |
| illustration | You can post something and go unnoticed for two days. So, in that aspect, it [...] felt almost a little counterproductive [...]it just didn't necessarily help with the isolation. [ID 05, aged 22 years] |
| Finding119 | banning discussions about suicide could be challenging for people who need to talk about their suicidality. **(U)** |
| illustration | I know for myself sometimes when you want to - you just need to say something and to some people it's going to sound really bad but you're genuinely like I just need to get this out of my head. [ID 08, aged 22 years] |
| Study ID: Rebecca Grist2018 | |
| Finding120 | This accessibility and variety were valued by young people. **(U)** |
| illustration | I’ve found it really helpful because I’ve tried using other apps and stuff but they only really cover like one aspect of what BlueIce offers...having app where there’s everything that you need like a little tool kit I think that’s really helpful [Participant number 142] |
| Finding121 | This privacy was reported to be a significant benefit of the app. **(U)** |
| illustration | It’s perfectly discreet and like it doesn’t’ have to be something that has to be hidden...it’s just, I like the design of it, I think it’s very well designed [Participant number 111] |
| Finding122 | they would like the ability to personalize BlueIce even more. **(U)** |
| illustration | Being able to personalise the colour, I thought that would be quite cool. [Participant number 113] |
| Finding123 | This ease, accessible and simple of use was valued by participants as they did not want to add further stress to their situation. **(U)** |
| illustration | t was really easy to get into and start using if you know what I mean, it was like once you knew how to use it, it was really easy. [Participant number 126] |
| Finding124 | BlueIce was a place where thoughts and feelings that were too difficult to share with others could be externalized. **(U)** |
| illustration | I really liked the diary ‘cos...I dunno, ‘cos especially when I’m feeling down I’m like ‘OH I’m always so sad’ or ‘what is the point’ but actually if I look back to the diary I can see that there were days when I was happy. [Participant number 135] |
| Finding125 | there were times when they are open to receiving support to stop self-harming and other times when they were less willing to accept help and to stop the act of self-harm. **(U)** |
| illustration | I might just sort of feel a bit wilful, sort of, somewhat want to stop myself from self-harming but sometimes I just want to self-harm and that’s the end of it really [Participant number 120] |
| Finding126 | This generally involved an increased need for more face-to-face contact with CAMHS, which took precedence over using BlueIce. **(U)** |
| illustration | I was more in contact with the crisis team and more involved with like CAMHs like more than once a week so I was kind of getting more things to do than just use the BlueIce app [Participant number 137] |
| Finding127 | Not being able to access BlueIce on their own device was a significant barrier to engagement for those participants who were provided an Android phone. **(C)** |
| illustration | summary by author |
| Study ID: Ana Radovic 2021 | |
| Finding128 | Adolescents, in particular, thought that they and their peers would be unlikely to bring it up on their own but might disclose if asked. **(U)** |
| illustration | A lot of the time maybe people are suffering from mental health problems may not want to talk about it outright and may not bring it up, so if they give you like a survey or something that might help kind of encourage them to talk about it more.” [adolescent] |
| Finding129 | Some adolescents described not filling out depression screenings accurately in the past out of a desire to avoid treatment. **(U)** |
| illustration | “I wasn’t completely honest when I first filled out like the tablet [...] because I didn’t want to get treatment. [...] Like if they asked me if my mood was like 1 through 10, say it was like a 1 I said it was like a 3 or 4.” [adolescent] |
| Finding130 | adolescents sometimes described not wanting to be honest in screening for fear of others finding out that they have mental health problems. **(U)** |
| illustration | “Fear of it getting out, like if their parents were to find out. I know that it’s harder to open up if you think more people are going to know about it cause it feels like people are judging you.” [adolescent on why adolescents may be dishonest in screening] |
| Finding131 | Adolescents sometimes described feeling more comfortable disclosing depression in a computerized screening. **(U)** |
| illustration | “When you’re talking to a person, you’re thinking about what they’re thinking, but when you’re talking to a computer, you know it’s not, like, judging you or having any thoughts of its own. It’s just recording what you’re inputting.” [adolescent] |
| Finding132 | adolescents, or providers did not want SW to replace face-to-face discussions of screening results and mental health between providers and patients. **(U)** |
| illustration | “[Screening Wizard should be used] when you go in for like a checkup or something, see like um how the person’s feeling and if they are feeling bad maybe you can um talk to them about it during the checkup or something like that.” [adolescent] |
| Study ID: Joseph Tighe 2020 | |
| Finding133 | it provided a service that was accessible when they needed it. **(U)** |
| illustration | Accessible when you feel like shit and you need to find a way to de-stress. It’s very helpful. [27-F-11] |
| Finding134 | may have been reluctant or afraid to speak to family members or health care professionals in a face-to-face setting could still access support. **(U)** |
| illustration | ...it’s non-judgmental. Biggest stigma and shame are being judged by a counsellor or anyone. Can sit in a room on your own and do it. [26-F-8] |
| Finding135 | the privacy offered by the app was valued. **(U)** |
| illustration | “Less worrying than actually talking to someone” [27-M-2]. |
| Finding136 | when creating an app that is accessible to vulnerable people who may not have access to any other support — an app may simply not be enough in the face of strong emotions. **(U)** |
| illustration | If really remote [users] may feel isolated, may feel they have no one to confide in. Risk of invoking feelings, no follow up. [23-F-13] |
| Finding137 | Some participants could see the benefit of using the app as an educational tool that improved mental health literacy, thereby facilitating improved cross-cultural communicate-on with mental health professionals. **(U)** |
| illustration | ...Sometimes you can’t find the words. Hearing other stories, makes it easier to find those words. Sometimes you say nothing to clinicians, but the app educates [on the] language to use. [27-M-2] |
| Finding138 | the app’s activities helped to improve self-awareness and interpersonal communications, which also spoke to ideas around improving cross-cultural communication. **(U)** |
| illustration | ...even to recognize that you are not quite right ... encourages you to have a conversation with yourself. Activities to improve behavior and interactions with other people. [23-F-13] |
| Finding139 | This may indicate the usefulness of the app for brief interventions rather than for intensive therapeutic use. **(U)** |
| illustration | If you use the app when distressed and impulsive; then, that reduces the depression and suicidal thinking. I took it to [location away from home]; shit things happened, and it helped. [19-F-1] |
| Study ID: Mareka Frost 2016 | |
| Finding140 | Young people reported a need for advice on how to help themselves, suggestions for reducing self-injury, or strategies to avoid self-injury. **(U)** |
| illustration | Ideas on what to do instead of self-harming, or what to do when the thought comes across your mind. |
| Finding141 | A desire for personalized support from both professionals and peers emerged. **(U)** |
| illustration | Someone that is real and that I can talk to. |
| Finding142 | Young people identified a general desire for understanding and a specific desire to know others had a shared experience. **(U)** |
| illustration | Community feeling – not just facts and figures. I want to feel like there are other people experiencing this, and how they got/ get through it. But at the same time, I want personal help. I want someone to understand my situation. |
| Finding143 | Many young people specifically identified a need for information about self-in-jury, research, statistics, and fact sheets and highlighted the importance of relevance and reliability of such information. **(U)** |
| illustration | Being able to find information that I am too scared to ask for. |
| Finding144 | The need for appropriate online culture in online support services was endorsed almost as frequently, need for a nonjudgmental, safe environment and interactions. **(U)** |
| illustration | that it acknowledges that self-harm is sometimes a survival strategy. That it does not stigmatize self-harm, blame people who self-harm, or ignore the underlying causes of self-harm. |
| Finding145 | Safety in online services for self-injury centered around the need for moderation. **(U)** |
| illustration | That it is safe and not people just talking graphically about how they self-harm or flaming others or triggering others. |
| Finding146 | when in crisis, free services with instant access and real-time support, no wait, available anywhere and accessible. **(U)** |
| illustration | Being able to access online counselling on mobile Internet. |
| Finding147 | need for immediate support without long waiting periods as particularly important in providing online support for self-injury. **(U)** |
| illustration | That the people can talk straight away and you wouldn’t have to wait for over 5 min to talk to a professional. |
| Finding148 | The majority of responses related to anonymity and confidentiality. **(U)** |
| illustration | That I have the opportunity to remain anonymous. Anonymity is something that is very important to me, especially in relation to such a private and personal topic such as self-harm. I would not use an online support service to talk about self-harm if I did not have the option to remain anonymous. |
| Finding149 | The need for assistance with help-seeking identifying as important. **(U)** |
| illustration | Advice on how to seek help from my GP and bring up the subject with family/partner. |
