# Supplementary figures and images for: Needs for mobile and internet-based psychological intervention in patients with self-injury and suicide-related behaviors: a qualitative systematic review
Source: BMC Psychiatry. 2024 Jan 4;24:26. doi: 10.1186/s12888-023-05477-2 (PMC10768375; doi:10.1186/s12888-023-05477-2)

Additional file 3: One of the themes of qualitative synthesis: Needs in the perception link

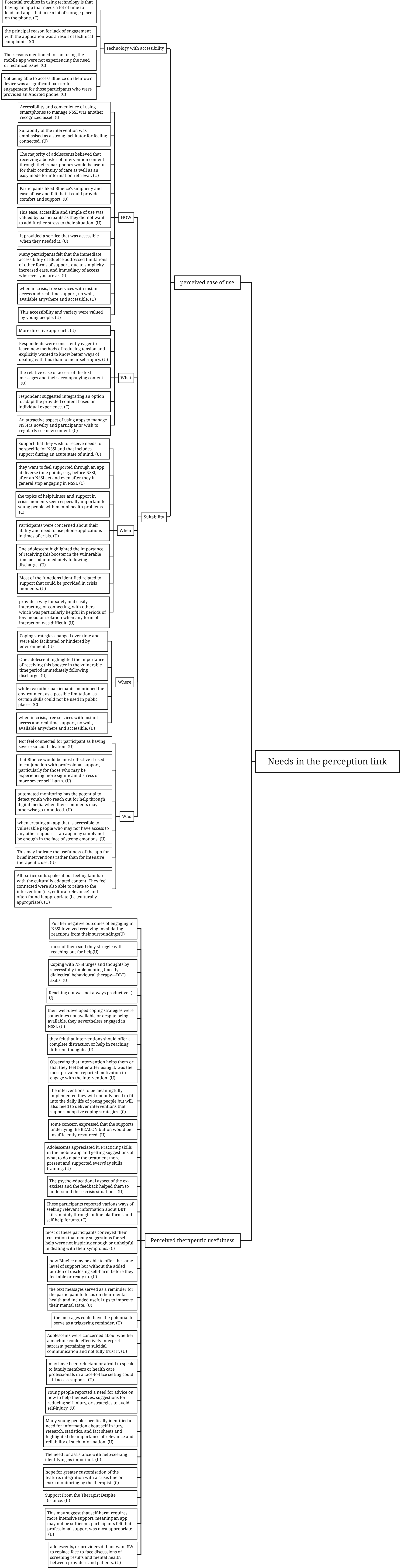

Supplement: Supplementary file 3 — Additional file 3. One of the themes of qualitative synthesis: Needs in the perception link. [file 12888_2023_5477_MOESM3_ESM.pdf]

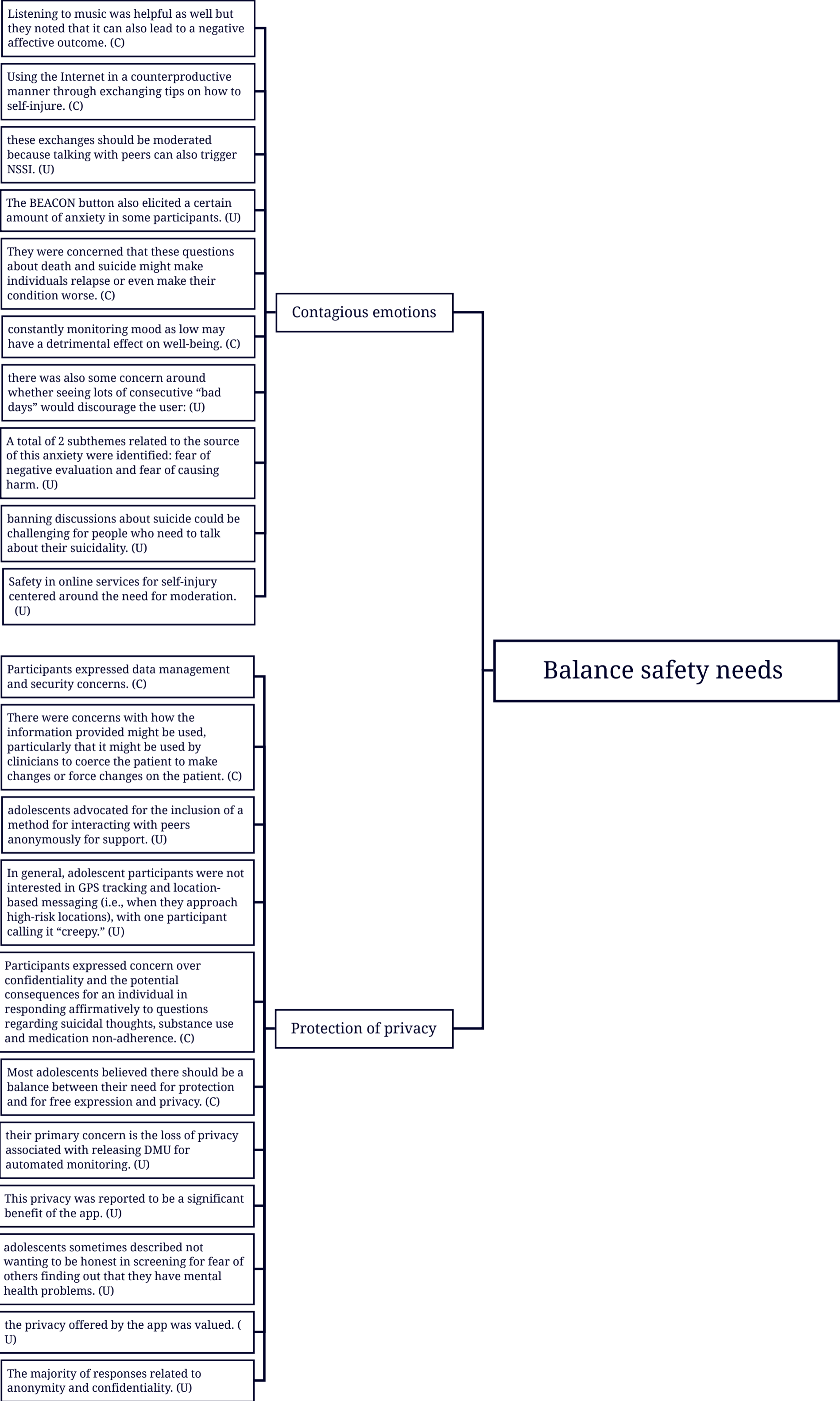

Supplement: Supplementary file 4 — Additional file 4. One of the themes of qualitative synthesis:Balance safety needs. [file 12888_2023_5477_MOESM4_ESM.pdf]

Additional file 5: One of the themes of qualitative synthesis: Advanced needs

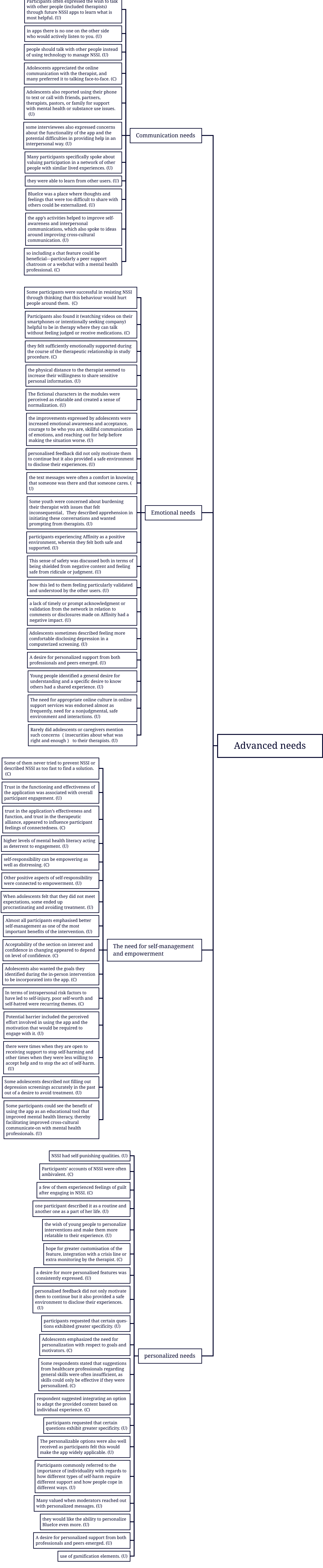

Supplement: Supplementary file 5 — Additional file 5. One of the themes of qualitative synthesis: Advanced needs. [file 12888_2023_5477_MOESM5_ESM.pdf]
