# Supplementary material for: Needs for mobile and internet-based psychological intervention in patients with self-injury and suicide-related behaviors: a qualitative systematic review
Source: BMC Psychiatry. 2024 Jan 4;24:26. doi: 10.1186/s12888-023-05477-2 (PMC10768375; doi:10.1186/s12888-023-05477-2)
Supplement: Supplementary file 6 — Additional file 6. The results of the GRADE-CERQual of the synthesized findings. [file 12888_2023_5477_MOESM6_ESM.docx]

| **Additional file 6: The results of the GRADE-CERQual of the synthesized findings** | | | |
| --- | --- | --- | --- |
| **Summary of qualitative findings** | | | |
| **Summary of review finding** | **Studies contributing to the review finding** | **CERQual assessment (confidence in the findings)** | **Explanation of CERQual assessment** |
| Technology with accessibility | Anja Cuš ˇ 2021; Craig Mackie 2017; Olivia Simonsson 2021; Rebecca Grist2018 | Moderate confidence | 1. 2 articles were post-hoc interviews and may have been biased. 2. 4 articles covering the European region only, with limited coverage. |
| HOW Suitability | Anja Cuš ˇ 2021; Ozlem Eylem 2021; McManama O’Brien 2019; Bethany Cliffe 2022; Rebecca Grist2018; Joseph Tighe 2020; Mareka Frost 2016 | High confidence | 1. One article did not mention whether the interviewer-interviewee relationship was taken into account. There were 2 articles with possible recall bias in the data collection. 2. 5 articles covering the European region only, with limited coverage. |
| What Suitability | Ozlem Eylem 2021; Tobias Schiffler 2022; Natasha Josifovski 2022; Rebecca Grist2018; Anja Cuš ˇ 2021 | Moderate confidence | 1. 2 articles did not mention whether the interviewer-interviewee relationship was taken into account. 2. 5 articles covering the European region only, with limited coverage. |
| When Suitability | Anja Cuš ˇ 2021; Craig Mackie 2017; McManama O’Brien 2019; Bethany Cliffe 2022; E. Baileyy 2021 | Moderate confidence | 1. 1 article did not allow participants to co-check transcription results. 2. 5 articles covering Europe only, limited coverage. |
| Where Suitability | Anja Cuš ˇ 2021; McManama O’Brien 2019; Tobias Schiffler 2022; Mareka Frost 2016 | Moderate confidence | 1. One article did not mention whether the interviewer-interviewee relationship was taken into account 2. 5 articles covering Europe only, limited coverage |
| Who Suitability | Ozlem Eylem 2021; Bethany Cliffe 2022; Candice Biernesser 2021; Joseph Tighe 2020; | Low confidence | 1. Two articles did not mention whether the interviewer-interviewee relationship was taken into account, and one article had a data collection lag. 2. 4 articles covering Europe only, limited coverage |
| Personalization | Anja Cuš ˇ 2021; Craig Mackie 2017; Ozlem Eylem 2021; J. Kasckow 2014; McManama O’Brien 2019; Tobias Schiffler 2022; J. Kasckow 2015; Bethany Cliffe 2022; E. Baileyy 2021; Rebecca Grist2018; Mareka Frost 2016 | High confidence | 1. Four articles did not mention whether the interviewer-interviewee relationship was taken into account. 2. One article collected with possible bias and one article did not allow participants to co-check transcription results. |
| Contagious emotions | Anja Cuš ˇ 2021; Craig Mackie 2017; J. Kasckow 2014; Bethany Cliffe 2022; E. Baileyy 2021; Mareka Frost 2016 | High confidence | 1. One article did not allow participants to co-check transcription results, and two articles did not mention whether the interviewer-interviewee relationship was taken into account. |
| Protection of privacy | Anja Cuš ˇ 2021; J. Kasckow 2014; McManama O’Brien 2019; J. Kasckow 2015; Candice Biernesser 2021; Rebecca Grist2018; Ana Radovic 2021; Joseph Tighe 2020; Mareka Frost 2016 | Moderate confidence | 1. Three articles did not mention whether the interviewer-interviewee relationship was taken into account, and two may have had a lag in data collection. |
| Communication needs | Anja Cuš ˇ 2021; Olivia Simonsson 2021; McManama O’Brien 2019; Tobias Schiffler 2022; E. Baileyy 2021; Rebecca Grist2018; Joseph Tighe 2020 | High confidence | 1. 2 articles had possible data collection recall bias and 1 article did not mention whether the interviewer-interviewee relationship was taken into account |
| Emotional needs | Anja Cuš ˇ 2021; Craig Mackie 2017; Olivia Simonsson 2021; Ozlem Eylem 2021; Natasha Josifovski 2022; Candice Biernesser 2021; E. Baileyy 2021; Ana Radovic 2021; Mareka Frost 2016 | Moderate confidence | 1. Four articles did not mention whether the interviewer-interviewee relationship was taken into account; one article had a possible data collection recall bias; and one article did not allow participants to co-check transcription results. |
| Therapy needs | Anja Cuš ˇ 2021; Craig Mackie 2017; Olivia Simonsson 2021; Ozlem Eylem 2021; Tobias Schiffler 2022; Bethany Cliffe 2022; Natasha Josifovski 2022; Candice Biernesser 2021; Joseph Tighe 2020; Mareka Frost 2016; Ana Radovic 2021 | High confidence | 1. 3 articles did not mention whether the interviewer-interviewee relationship was taken into account. 2. 1 article had a data collection lag and 1 article may have had a data collection recall bias. |
| The need for self-management and empowerment | Anja Cuš ˇ 2021; Craig Mackie 2017; Olivia Simonsson 2021; Ozlem Eylem 2021; McManama O’Brien 2019; Tobias Schiffler 2022; Bethany Cliffe 2022; Rebecca Grist2018; Ana Radovic 2021; Joseph Tighe 2020. | High confidence | 1. The article may have suffered from data collection recall bias. 2. The article does not mention whether the interviewer-interviewee relationship was taken into account. |

**The results of the GRADE-CERQual**

| **finding** | **Methodological limitation** | **Coherence** | **relevance** | **Sufficiency of data** | **CERQual** |
| --- | --- | --- | --- | --- | --- |
| Technology with accessibility | 2 high quality articles  2 moderate quality articles | High | 3 articles closely related  1 article moderately relevant | Overall: Moderate confidence  covering 4 countries | Moderate confidence |
| HOW Suitability | 4 high quality articles  3 moderate quality articles | High | 6 articles closely related  1 article moderately relevant | Overall: Moderate confidence  covering 5 countries | High confidence |
| What Suitability | 2 high quality articles  3 moderate quality articles | High | 5 articles closely related | Overall: Moderate confidence  covering 4 countries | Moderate confidence |
| When Suitability | 4 high quality articles  1 moderate quality article | High | 2 articles closely related  3 article moderately relevant | Overall: Moderate confidence  covering 4 countries | Moderate confidence |
| Where Suitability | 3 high quality articles  1 moderate quality article | Moderate | 3 articles closely related  1 article moderately relevant | Overall: Moderate confidence  covering 4 countries | Moderate confidence |
| Who Suitability | 2 high quality articles  2 moderate quality articles | Moderate | 3 articles closely related  1 article moderately relevant | Overall: Moderate confidence  covering 3 countries | Low confidence |
| Personalization | 5 high quality articles  6 moderate quality articles | High | 6 articles closely related  5 article moderately relevant | Overall: Moderate confidence  covering 7 countries | High confidence |
| Contagious emotions | 3 high quality articles  3 moderate quality articles | High | 3 articles closely related  3 article moderately relevant | Overall: Moderate confidence  covering 5 countries | High confidence |
| Protection of privacy | 4 high quality articles  5 moderate quality articles | Moderate | 4 articles closely related  5 article moderately relevant | Overall: Moderate confidence  covering 5 countries | Moderate confidence |
| Communication needs | 4 high quality articles  3 moderate quality articles | High | 5 articles closely related  2 article moderately relevant | Overall: Moderate confidence  covering 5 countries | High confidence |
| Emotional needs | 3 high quality articles  6 moderate quality articles | High | 5 articles closely related  4 article moderately relevant | Overall: Moderate confidence  covering 6 countries | Moderate confidence |
| Therapy needs | 6 high quality articles  5 moderate quality articles | High | 9 articles closely related  2 article moderately relevant | Overall: Moderate confidence  covering 7 countries | High confidence |
| The need for self-management and empowerment | 7 high quality articles  3 moderate quality articles | High | 7 articles closely related  3 article moderately relevant | Overall: Moderate confidence  covering 7 countries | High confidence |
